# Trends in Socioeconomic Inequalities and Prevalence of Anemia Among Children and Nonpregnant Women in Low- and Middle-Income Countries

**DOI:** 10.1001/jamanetworkopen.2018.2899

**Published:** 2018-09-28

**Authors:** Fan Yang, Xueyi Liu, Panpan Zha

**Affiliations:** 1 School of Health Policy and Management, Nanjing Medical University, Nanjing, China

## Abstract

**Importance:**

Anemia remains a major challenge for women’s and children’s health in low- and middle-income countries (LMICs).

**Objective:**

To assess the socioeconomic inequalities and prevalence of anemia among children and nonpregnant girls and women in LMICs over time.

**Design, Setting, and Participants:**

This cross-sectional and repeated cross-sectional analysis used data from the Demographic and Health Surveys collected from January 1, 2000, through December 31, 2014. Initial cross-sectional data sets constructed from the most recent surveys included 163 419 children aged 6 to 59 months and 304 202 nonpregnant girls and women aged 15 to 49 years (hereinafter referred to as nonpregnant women) from 45 LMICs. Repeated cross-sectional data sets from the most recent and the earliest surveys consisted of 322 088 nonpregnant women from 25 LMICs and 182 273 children from 24 LMICs. Analyses were conducted from June 1, 2016, through July 3, 2018.

**Main Outcomes and Measures:**

Total and severe anemia for children aged 6 to 59 months (hemoglobin level, <11 and <7 g/dL, respectively) and nonpregnant women aged 15 to 49 years (<12 and <8 g/dL, respectively) were defined according to the World Health Organization hemoglobin cutoff levels. The slope index of inequality (SII) and the relative index of inequality (RII) were calculated to determine anemia inequalities.

**Results:**

Among the 163 419 children aged 6 to 59 months and the 304 202 nonpregnant women aged 15 to 49 years in the initial cross-sectional data sets, 34 of 45 countries had anemia prevalence levels for children greater than 40% and 37 of 45 had anemia prevalence levels greater than 20% for nonpregnant women. Among the 182 273 children from 24 LMICs and 322 088 nonpregnant women from 25 LMICs in the repeated cross-sectional data sets, the annualized absolute decreases in the prevalence of anemia ranged from 0.67 to 3.98 percentage points in 16 of the 24 LMICs; for cases of severe anemia, the decrease was 0.03 to 0.82 percentage points in 15 LMICs. Among 322 088 nonpregnant women from 25 LMICs in the repeated cross-sectional data sets, the annualized absolute decreases in the prevalence of anemia ranged from 0.49 to 2.59 percentage points in 17 of 25 LMICs; for severe anemia, the decrease was 0.03 to 0.29 percentage points in 15 LMICs. The SII was significantly negative and the RII was significantly less than 1 in 37 of the 45 LMICs among children and in 26 of the 45 LMICs for nonpregnant women. The annualized changes in the SII approached 0 in 16 of the 24 LMICs among children and in 11 of 25 LMICs among nonpregnant women.

**Conclusions and Relevance:**

Most LMICs continue to exhibit a high prevalence of anemia among children and nonpregnant women, although the prevalence of total and severe anemia have decreased in many LMICs. Anemia inequalities among children and nonpregnant women persist in most LMICs.

## Key Points

**Question:**

How did anemia inequalities change over time in low- and middle-income countries?

**Findings:**

In this cross-sectional study of 163 419 children aged 6 to 59 months and 304 202 nonpregnant girls and women aged 15 to 49 years, the socioeconomic inequalities of anemia did not decrease among children in approximately 80% of low- and middle-income countries. For nonpregnant women, that figure was approximately 60% of low- and middle-income countries.

**Meaning:**

To reduce the anemia inequalities found in most low- and middle-income countries, future efforts should attend to policies designed to reach vulnerable groups, specifically those with lower socioeconomic status.

## Teaser

This cross-sectional analysis uses data from the Demographic and Health Surveys to assess the socioeconomic inequalities and prevalence of anemia among children and nonpregnant women in low- and middle-income countries.

## Introduction

Anemia remains a major challenge for the health and development of women and children in low- and middle-income countries (LMICs).^1,2,3,4^ Anemia is a serious public health problem affecting 293.1 million children and 468.4 million nonpregnant women in LMICs.^1,3,4^ In most LMICs, anemia is a major cause of child and maternal mortality^1,2^ and is associated with an increased risk of low birth weight, cognitive impairment,^3^ increased susceptibility to infection,^1^ and delayed physical and mental development with diminished ability to work.^1,3^ Significantly, anemia serves as an indicator of socioeconomic disadvantage because it is inversely related to the socioeconomic status of households in developing settings^1,3,4^; individuals with low socioeconomic status are at a higher risk of exposure to anemia and its sequelae.^3^ Moreover, both the prevalence of anemia and health equity of children and nonpregnant women in many LMICs represent important aspects of the Countdown to 2015.^5,6^ Research on the prevalence of anemia and the health equity of children and nonpregnant women in LMICs is crucial for the development of new interventions to combat anemia.

Although previous studies have estimated the magnitude of anemia among children and nonpregnant women,^4,7,8,9^ few systematic attempts have been made to monitor the socioeconomic inequalities of anemia and how anemia inequalities vary over time in LMICs. Furthermore, anemia inequalities have not been explored for comparison across countries^10^ because standardized measurement tools and similar surveys have not been used. Therefore, we used data from the Demographic and Health Surveys (DHS) to systematically evaluate the socioeconomic inequalities and prevalence of anemia among children and nonpregnant women in LMICs over time.

## Methods

### Data Sources

We conducted cross-sectional and repeated cross-sectional analyses with data from the DHS.^11,12,13^ Our study followed the Strengthening the Reporting of Observational Studies in Epidemiology (STROBE) reporting guideline. The DHS protocols and the process for obtaining oral informed consent for this study from all participants were approved by the institutional review board of ICF International, Calverton, Maryland.

The DHS consists of household surveys designed to collect data on sociodemographic characteristics, child and maternal health, family planning, malaria, nutrition, sanitary conditions, and HIV and/or AIDS in 85 LMICs; 57 of these countries conducted at least 2 surveys.^14,15,16^ Multiple surveys allow for repeated cross-sectional analyses that could systematically evaluate trends in the socioeconomic inequalities and prevalence of anemia. Therefore, we constructed initial and repeated cross-sectional data sets using women’s and/or children’s surveys from the DHS from January 1, 2000, through December 31, 2014, excluding countries where no surveys were conducted. Four data sets were selected for inclusion in the study (eFigure 1 in the Supplement), and all analysis was limited to nonpregnant girls and women aged 15 to 49 years (hereinafter referred to as nonpregnant women) and children aged 6 to 59 months (eMethods 1 in the Supplement).

The first and second data sets were developed from cross-sectional surveys across countries where at least 1 survey had been conducted. Data from the most recent surveys that captured hemoglobin concentrations and sociodemographic characteristics for children aged 6 to 59 months and nonpregnant women aged 15 to 49 years were selected to construct the data set. The first cross-sectional data set included children aged 6 to 59 months developed from the most recent surveys of 45 LMICs, with the final number of analytic participants totaling 163 419 children aged 6 to 59 months (eFigure 1 in the Supplement). The second cross-sectional data set included nonpregnant women aged 15 to 49 years developed from the most recent surveys of 45 LMICs, with the final number of analytic participants totaling of 304 202 nonpregnant women aged 15 to 49 years (eFigure 1 in the Supplement). These 2 cross-sectional data sets were used to conduct cross-sectional analyses examining the socioeconomic inequalities and prevalence of anemia among children and nonpregnant women.

The third and fourth data sets were developed from repeated cross-sectional surveys that included countries where at least 2 surveys were completed and that captured hemoglobin concentrations and sociodemographic characteristics of children aged 6 to 59 months and nonpregnant women aged 15 to 49 years. If a country had at least 2 data sets from the DHS available, the data of the earliest and most recent surveys were selected to construct the data set. Therefore, we included the earliest and the most recent surveys of the 24 LMICs in the third cross-sectional data set for children aged 6 to 59 months, a total of 182 273 analytic participants (eFigure 1 in the Supplement). We included the earliest and the most recent surveys of the 25 LMICs in the fourth cross-sectional data set, a total of 322 088 nonpregnant women (eFigure 1 in the Supplement). These third and fourth cross-sectional data sets were used to conduct the repeated cross-sectional analyses that examined the trend in the socioeconomic inequalities and prevalence of anemia among children and nonpregnant women over time. The years of the surveys as well as the sample sizes are provided in the eTables 1 to 4 in the Supplement.

### Measures and Selection of Indicators

The DHS used standardized protocols and procedures to obtain data on hemoglobin concentrations.^17^ Following World Health Organization guidelines, total anemia was defined as a hemoglobin level less than 11 g/dL for children aged 6 to 59 months and less than 12 g/dL for nonpregnant women aged 15 to 49 years (to convert hemoglobin to grams per liter, multiply by 10.0). Severe anemia for children aged 6 to 59 months was defined as a hemoglobin level less than 7 g/dL, whereas severe anemia was defined as a hemoglobin level less than 8 g/dL for nonpregnant women aged 15 to 49 years.^9,18^

The DHS data treated the distribution of wealth in association with other households.^13,15,19^ The DHS wealth index was based on a set of variables related to residence characteristics, the household ownership of selected assets, and the availability of basic community-level services (eg, water and electricity).^15,19^ The wealth index is commonly used in the systematic comparison of health inequalities^10,12,13,15,20,21,22^ and closely matches indicators used to determine household socioeconomic status in LMICs.^19^ The DHS breaks down the wealth index into 5 quintiles.

### Statistical Analysis

Data were analyzed from June 1, 2016, through July 3, 2018. All estimates used to determine anemia were weighted and proved appropriate for the DHS complex survey design. Estimates of the prevalence of total and severe anemia among nonpregnant women were adjusted using world population data obtained from the World Health Organization. We used linear regression models to estimate annual absolute changes in the prevalence of total and severe anemia with 95% CIs, adjusted for age and survey time, and we took the earliest survey time as the reference group. The changes in the prevalence of total and severe anemia were measured by the annual absolute changes in percentage points and were estimated through a calculation of the difference between anemia prevalence in the earliest and most recent surveys divided by the number of years between these 2 surveys.

The slope index of inequality (SII) and the relative index of inequality (RII) were calculated to determine the absolute and relative socioeconomic inequalities of anemia, respectively.^20,21,22,23,24^ The SII and RII can be interpreted as the prevalence rate difference and the prevalence rate ratio, respectively; they are more complex than the rate difference and ratio that only measure the highest and the lowest quintiles. Moreover, because the SII and RII allow for socioeconomic inequalities across the entire socioeconomic distribution to be summarized in the index, they promote comparison of anemia inequalities across countries and over time.^20,21,22^ The children and nonpregnant women in this study were separately ranked from the lowest (rank 0) to the highest (rank 1) wealth to estimate their positions in the cumulative distribution of socioeconomic status.^15^ The SII can be interpreted as the expected difference between the prevalence of anemia at ranks 1 and 0 (across the entire socioeconomic distribution).^25^ In this study, the SII was calculated using linear regression and was equal to the value of the slope of the line regression that estimates the absolute effect on the prevalence of anemia, ranging from ranks 1 to 0.^25^ The RII is estimated as the expected ratio between the prevalence of anemia at ranks 1 and 0 (across the entire socioeconomic distribution). The RII was obtained by logistic regression and was equal to the slope of the logistic regression that estimates relative effect on the prevalence of anemia, ranging from ranks 1 to 0.^25^ An SII greater than 0 and an RII greater than 1 would indicate that individuals with lower socioeconomic status would be more likely to have anemia, whereas the reverse inequality would indicate lower anemia prevalence among populations with lower socioeconomic status.^12,22^

The annualized changes of the SII and RII were calculated to adjust for the interval of time between the earliest and most recent surveys.^13,21,22,25^ With the use of linear regression and logit models, changes in SII and RII were estimated with 95% CIs, taking the earliest survey time as the reference group and adjusting the survey time. Changes in the SII were measured by the annual absolute change, expressed by dividing the difference between the SII in the earliest and the most recent surveys by the number of years between the 2 surveys. The relative scale of the change in RII was estimated through a calculation of percentage change in RII between the earliest and most recent surveys divided by the number of years between the 2 surveys. Positive values for the annualized change of the SII and RII indicate a reduction in inequality, whereas negative values indicate an increase in inequality. As shown in eTables 7 to 12 in the Supplement, we also examined anemia inequalities for other dimensions of socioeconomic position, including educational level (or maternal educational level in the case of children), using the same analysis method (eMethods 2 in the Supplement). A 2-sided *P* < .05 was regarded as statistically significant. All statistical tests were 2-tailed and were performed using Stata software (version 12; StataCorp).

## Results

The cross-sectional data sets included 163 419 children aged 6 to 59 months and 304 202 nonpregnant women aged 15 to 49 years (Table). The weighted prevalence of total anemia and severe anemia among children in LMICs was 55.32% (95% CI, 54.85%-55.80%; 91 462 participants) and 2.81% (95% CI, 2.68%-2.94%; 4691 participants), respectively. The weighted prevalence of total and severe anemia among nonpregnant women was 34.89% (95% CI, 34.52%-35.27%; 106 686 participants) and 0.58% (95% CI, 0.55%-0.62%; 1871 participants), respectively. The prevalence of total and severe anemia proved highest among the lowest socioeconomic groups (59.80% [95% CI, 59.01%-60.58%] and 3.59% [95% CI, 3.33%-3.86%], respectively, for children aged 6-59 months; 37.58% [95% CI, 36.88%-38.27%] and 0.78% [95% CI, 0.68%-0.87%], respectively, for nonpregnant women aged 15-49 years) and lowest in the highest socioeconomic groups (47.46% [95% CI, 46.42%-48.52%] and 1.44% [95% CI, 1.23%-1.65%], respectively, for children aged 6-59 months; 34.42% [95% CI, 31.70%-33.14%] and 0.46% [95% CI, 0.40%-0.52%], respectively, for nonpregnant women aged 15-49 years). The highest absolute inequality across the entire socioeconomic distribution was total anemia among children. For example, the SIIs for total and severe anemia among children were −14.66 (95% CI, −16.07 to −13.26) and −2.59 (95% CI, −2.98 to −2.20), respectively. Evidence existed of greater inequality with respect to anemia among children than in nonpregnant women (the RII for severe anemia was 0.38 [95% CI, 0.33-0.44] for children and 0.61 [95% CI, 0.53-0.72] for nonpregnant women; for total anemia, 0.55 [95% CI, 0.52-0.58] for children and 0.77 [95% CI, 0.75-0.79] for nonpregnant women).

**Table.** Total and Severe Anemia Prevalence for Each Socioeconomic Position Group and Anemia Inequalities Among Nonpregnant Women and Children in 45 Low- and Middle-Income Countries at the Most Recent Survey^a^

Variable	Children Aged 6-59 mo					Nonpregnant Women Aged 15-49 y				
	Total No. of Participants	Anemia		Severe Anemia		Total No. of Participants	Anemia		Severe Anemia	
		No. of Participants	Weighted Prevalence, % (95% CI)	No. of Participants	Weighted Prevalence, % (95% CI)		No. of Participants	Weighted Prevalence, % (95% CI)	No. of Participants	Weighted Prevalence, % (95% CI)
Total	163 419	91 462	55.32 (54.85 to 55.80)	4691	2.81 (2.68 to 2.94)	304 202	106 686	34.89 (34.52 to 35.27)	1871	0.58 (0.55 to 0.62)
Wealth										
Q1	42 496	25 335	59.80 (59.01 to 60.58)	1443	3.59 (3.33 to 3.86)	60 095	22 342	37.58 (36.88 to 38.27)	446	0.78 (0.68 to 0.87)
Q2	36 126	20 834	57.82 (56.01 to 58.64)	1139	3.17 (2.93 to 3.42)	58 380	21 057	36.06 (35.43 to 36.70)	395	0.66 (0.58 to 0.74)
Q3	32 493	18 287	55.76 (54.93 to 56.59)	1017	3.02 (2.78 to 3.26)	58 155	20 641	35.36 (34.71 to 36.01)	329	0.53 (0.45 to 0.60)
Q4	28 998	15 743	52.85 (51.95 to 53.74)	729	2.30 (2.07 to 2.53)	60 810	20 915	33.99 (33.35 to 34.62)	358	0.54 (0.47 to 0.61)
Q5	23 306	11 263	47.46 (46.42 to 48.52)	363	1.44 (1.23 to 1.65)	66 762	21 731	32.42 (31.70 to 33.14)	343	0.46 (0.40 to 0.52)
Rate difference in SII, percentage points (95% CI)^b^	NA	NA	−14.66 (−16.07 to −13.26)	NA	−2.59 (−2.98 to −2.20)	NA	NA	−5.91 (−6.49 to −5.33)	NA	−0.29 (−0.39 to −0.20)
RII, rate ratios (95% CI)^c^	NA	NA	0.55 (0.52 to 0.58)	NA	0.38 (0.33 to 0.44)	NA	NA	0.77 (0.75 to 0.79)	NA	0.61 (0.53 to 0.72)

Abbreviations: NA, not applicable; Q, quintile; RII, relative index of inequality; SII, slope index of inequality.

^a^ All estimates used to determine anemia were weighted and proved appropriate for the complex Demographic and Health Surveys design. Estimates of the prevalence of total and severe anemia among nonpregnant women were adjusted using world population data obtained from the World Health Organization.

^b^ Calculated to determine the absolute socioeconomic inequalities of anemia. An SII greater than 0 indicates that individuals with lower socioeconomic status would be more likely to have anemia, whereas the reverse inequality would indicate lower anemia prevalence among populations with lower socioeconomic status.

^c^ Calculated to determine the relative socioeconomic inequalities of anemia. An RII greater than 1 indicates that individuals with lower socioeconomic status would be more likely to have anemia, whereas the reverse inequality would indicate lower anemia prevalence among populations with lower socioeconomic status.

### Prevalence of Total and Severe Anemia Across Countries Over Time

eFigures 2 and 3 in the Supplement illustrate the prevalence of total and severe anemia among children and nonpregnant women in 45 LMICs at the time of the most recent survey. Substantial variations occurred among LMICs with regard to the prevalence of total and severe anemia. The weighted prevalence of total anemia ranged from 14.08% (3112 of 20 385) in Honduras in 2011 to 61.70% (4992 of 6760) in Yemen in 2013 for nonpregnant women and from 17.57% (254 of 1355) in Albania in 2008 to 87.89% (5239 of 5928) in Burkina Faso in 2010 for children. Weighted prevalence rates of severe anemia among nonpregnant women ranged from 0.02% (3 of 6464) in Egypt in 2014 to 2.64% (204 of 6760) in Yemen in 2013. The greatest prevalence of severe anemia among children was 15.49% (644 of 3754) in Yemen in 2013, whereas the lowest prevalence was 0 (0 of 1261) in Moldova in 2005. The prevalence of anemia in each of the LMICs is shown in eTables 1 and 2 in the Supplement.

eFigures 4 and 5 in the Supplement illustrate the annualized absolute changes in the prevalence of total and severe anemia among children from 24 LIMCs and nonpregnant women from 25 LMICs between the time of the earliest and the most recent surveys. In 16 of the 24 LMICs, the annualized absolute decrease in the prevalence of total anemia ranged between 0.67 percentage points (95% CI, −0.99 to −0.34 percentage points) in Ghana (2003-2014) and 3.98 percentage points (95% CI, −4.91 to −3.04 percentage points) in Madagascar (2003-2008) for children. However, 3 countries (Armenia, Bolivia, and Haiti) exhibited annualized absolute increases in the prevalence of total anemia among children. Fifteen of the 24 LMICs exhibited a significant decrease in the prevalence rates of severe anemia among children, ranging from 0.03 to 0.82 percentage points annually. Nevertheless, the rates in Bolivia increased annually by 0.30 percentage points (95% CI, 0.09-0.52 percentage points). For nonpregnant women, the prevalence of total anemia in 17 of 25 LMICs declined; annualized absolute changes ranged from 0.49 to 2.59 percentage points. However, the rates in Armenia increased annually by 2.34 percentage points (95% CI, 1.91-2.78 percentage points) and in Bolivia, by 0.93 percentage points (95% CI, 0.45-1.42 percentage points). In 15 of the 25 LMICs, the prevalence of severe anemia among nonpregnant women showed a significant decrease, ranging from 0.03 to 0.29 percentage points annually. The annualized absolute changes in the prevalence of anemia for children and nonpregnant women are shown in eTables 3 and 4 in the Supplement, respectively.

### Socioeconomic Inequalities in Anemia Across Countries and Time

**Figure 1.**
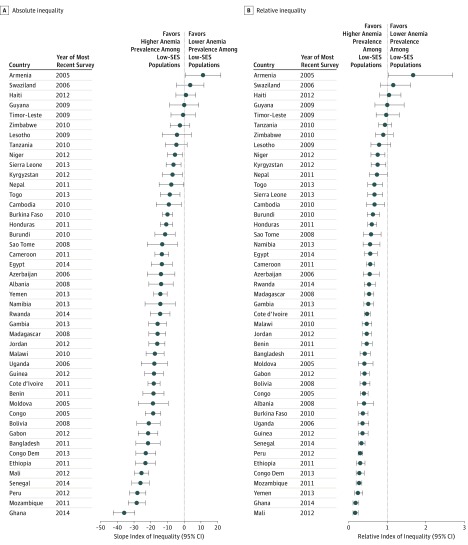
Absolute and Relative Inequality of Anemia Prevalence Among Children in Low- and Middle-Income Countries The absolute inequality (slope index of inequality) (A) and relative inequality (relative index of inequality) (B) are shown for prevalence of anemia among children aged 6 to 59 months for each low- and middle-income country. Error bars indicate 95% CIs. Specific numbers of participants, absolute and relative inequality values, and 95% CIs are provided in eTable 5 in the Supplement and omitted here for clarity. SES indicates socioeconomic status.

**Figure 2.**
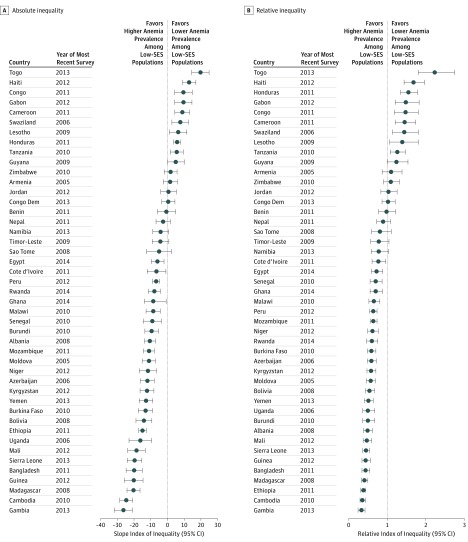
Absolute and Relative Inequality of Anemia Prevalence Among Nonpregnant Women in Low- and Middle-Income Countries The absolute inequality (slope index of inequality) (A) and relative inequality (relative index of inequality) (B) are shown for prevalence of anemia among nonpregnant women aged 15 to 49 years for each low- and middle-income country. Error bars indicate 95% CIs. Specific numbers of participants, absolute and relative inequality values, and 95% CIs are provided in eTable 6 in the Supplement and omitted here for clarity. SES indicates socioeconomic status.

Figure 1 and Figure 2 display the absolute and relative socioeconomic inequalities of total anemia among children and nonpregnant women for each of the LMICs at the time of the most recent survey, respectively. The SII and the RII were significantly less than 0 and less than 1, respectively, in 37 of the 45 LMICs among children. For example, the SII for Ghana was −35.96 (95% CI, −42.19 to −29.74), signifying that moving from the bottom to the top of the wealth distribution was associated with an estimated decrease of 35.96 cases of anemia per 100 children (eTable 5 in the Supplement). For nonpregnant women, the RII for total anemia was also statistically less than 1 in 26 of the 45 LMICs. For example, the RII for Ethiopia was 0.38 (95% CI, 0.33-0.43), indicating that the rate ratio was 38% between the prevalence of anemia for nonpregnant women at the top of the wealth distribution and for those at the bottom (across the entire socioeconomic distribution) (eTable 6 in the Supplement).

**Figure 3.**
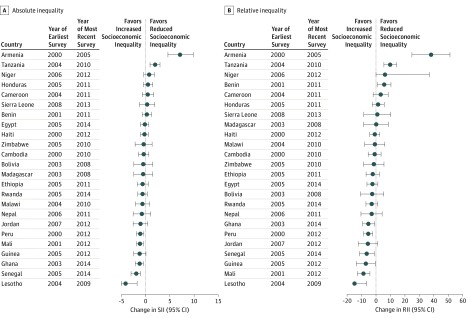
Annualized Changes of Absolute and Relative Inequality in Anemia Prevalence Among Children in Low- and Middle-Income Countries The annualized changes in absolute inequality (slope index of inequality [SII]) (A) and relative inequality (relative index of inequality [RII]) (B) for anemia prevalence among children aged 6 to 59 months are shown for each low- and middle-income country. Error bars represent 95% CIs. Specific numbers of participants, annualized changes of SII and RII values, and 95% CIs are provided in eTable 7 in the Supplement and omitted here for clarity.

**Figure 4.**
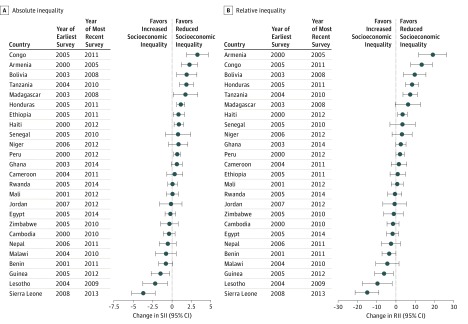
Annualized Changes of Absolute and Relative Inequality in Anemia Prevalence Among Nonpregnant Women in Low- and Middle-Income Countries The annualized changes in absolute inequality (slope index of inequality [SII]) (A) and relative inequality (relative index of inequality [RII]) (B) for anemia prevalence among nonpregnant women aged 15 to 49 years are shown for each low- and middle-income country. Error bars represent 95% CIs. Specific numbers of participants, annualized changes of SII and RII values, and 95% CIs are provided in eTable 8 in the Supplement and omitted here for clarity.

The annualized changes of the absolute and relative socioeconomic inequalities are separately displayed in Figure 3 and Figure 4. The annualized absolute changes in the SII were not significantly different from 0 in 16 of the 24 LMICs among children. However, 6 countries (Lesotho, Senegal, Ghana, Guinea, Mali, and Peru) showed significant absolute decreases in the SII for total anemia among children. Only the SIIs for Tanzania and Armenia exhibited annualized absolute increases for total anemia among children, indicating the reduction in socioeconomic inequalities (eTable 7 in the Supplement). The annualized absolute changes of the SII in 11 of the 25 LMICs were not significantly different from 0 among nonpregnant women. However, the SII for the countries of Sierra Leone, Lesotho, Guinea, and Benin represented annualized absolute decreases among nonpregnant women, indicating increases in socioeconomic inequalities (eTable 8 in the Supplement).

## Discussion

To our knowledge, this study is the first to systematically identify the magnitude of socioeconomic inequalities in the prevalence of anemia among children and nonpregnant women across LMICs and over time. The analysis showed that the prevalence rates decreased in 16 of 24 LMICs among children and 17 of 25 LMICs among nonpregnant women. More than 40% of children had anemia in 34 of 45 LMICs (76%) as well as 20% of the nonpregnant women in 37 of 45 LMICs (82%). Anemia was higher among populations with low socioeconomic status for nonpregnant women in 26 of 45 LMICs (58%) and for children in 37 of 45 LMICs (82%). Socioeconomic inequalities with regard to anemia did not decrease in approximately 80% of children in 16 of 24 LMICs and 60% of nonpregnant women in 11 of 25 LMICs.

The estimated total anemia prevalence of 55% among children in LMICs proved higher than the 43% anemia prevalence among children globally in 2011; the 35% among nonpregnant women aged 15 to 49 years also proved higher than the global estimate of 30%.^9^ In 2005, the UN System Standing Committee on Nutrition estimated the global prevalence of anemia to be 47% among children and 30% among nonpregnant women 15 to 49 years of age^7^; these numbers also were lower than the prevalence of total anemia identified in this study. The methods we used herein may produce fewer variations than the global estimates owing to the reliance on DHS data captured through uniform surveys as well as standardized collection and measurement tools across countries. In addition, the global estimates included data from high-income regions.^7,9^ More than 50% of nonpregnant women had anemia in some LMICs, including Yemen, Gabon, and Gambia. Approximately 80% of children were affected by anemia and about 10% were affected by severe anemia in some LMICs, with the highest rates exhibited in Burkina Faso, Yemen, and Mali. Studies have shown that iron deficiency may be the main cause of anemia in many developing countries.^3,26^ Other causes include malnutrition, parasitic infections, micronutrient deficiencies, and/or hereditary hemoglobinopathies.^1,3,27^ The magnitude of the problem among children and nonpregnant women demands urgent responses with interventions designed to address the causes of anemia in LMICs.

The annualized changes for children and nonpregnant women were more than 1 percentage point per year in approximately 50% of LMICs, whereas global anemia prevalence decreased by 0.16 percentage points per year from 1995 to 2000 for nonpregnant women and 0.36 percentage points per year for children.^28^ The findings also indicated that, although many LMICs had successfully addressed the problem of anemia among children and nonpregnant women, the positive changes in other countries have been very slow. For example, we found less improvement in Mali, where total and severe anemia among children reached approximately 80% and 10%, respectively. In Bolivia, the absolute anemia burden had not increased or decreased, but prevalence among children remained at approximately 60% and among nonpregnant women at nearly 40%. The high-risk groups (ie, children and women of childbearing age) did not receive adequate doses through dietary diversification and intake of foods with high iron bioavailability, alone or together with daily or intermittent iron supplements, along with other micronutrients because of the lack of attention being given to behavioral aspects associated with regular supplement use.^1,9^ These factors could be taken into account by clinical physicians to improve patient intakes. Anemia intervention in LMICs could focus on the effective delivery system of iron fortification and supplementation,^27^ take into account the diversity of cultures and food habits, and establish effective monitoring.^3,26^

A comparison of this research with previous studies proved difficult given that this study was the first, to our knowledge, to initiate a cross-country comparison over time. The results are consistent with other DHS analyses that examined maternal and child health services in LMICs.^12^ One previous study^12^ using DHS data demonstrated that coverage for all maternal, newborn, and child health interventions was considerably higher among the wealthiest quintiles. This study also found that the prevalence of anemia was 12% lower for children at the top of the wealth distribution than for those at the bottom. Countries that proved particularly relatively inequitable with respect to children with anemia were Mali, Ghana, Yemen, and Mozambique. Gambia, Cambodia, Ethiopia, and Madagascar were particularly inequitable with respect to nonpregnant women with anemia. Such inequalities may be due to the possibility that the individuals with low socioeconomic status are more likely to confront greater risk factors (eg, lack of health services,^29^ poor knowledge of nutrition, and poor diet^30^).

Nearly two-thirds of LMICs demonstrated a clear decline in anemia prevalence. However, the decline in anemia was not necessarily accompanied by a reduction in anemia inequality. The findings presented herein proved consistent with previous studies that showed that the global prevalence of stunted growth had decreased significantly from 1990 to 2011 but that the decrease was not necessarily accompanied by decreased inequalities with regard to its prevalence^22^: the poorest quintile consistently exhibited 2 times as much prevalence of stunted growth as the wealthiest quintile. Most intervention programs or policies have generally been designed to improve the overall health of the population. However, the beneficiaries of these policies have been more concentrated in sectors with higher levels of socioeconomic status.^31^

Some studies have shown that nutrition intervention policies improved nutritional quality (energy density decreased, vegetable and fruit quantities increased, and the mean adequate nutrition ratio increased). However, women with low socioeconomic status have shown little improvement; the policies did not reduce socioeconomic inequalities with regard to nutrition.^32,33^ Notably, the findings from this study showed that Senegal, Peru, Mali, Lesotho, Guinea, Ghana, and Sierra Leone demonstrated substantial increases in the absolute socioeconomic inequalities among children, nonpregnant women, or both. Some previous studies^13^ indicated that those who were at lower risk benefited more from the intervention policies than those who were previously at a higher risk. Successful intervention programs also removed financial barriers for maternal and newborn health services.^12,34^ Future efforts should devote greater attention to the policies designed to reach vulnerable groups with lower socioeconomic status as a means to reduce anemia inequalities.

### Limitations

Some limitations to this study merit attention. First, the DHS wealth index used as the socioeconomic indicator in the measurement of anemia inequalities was not an absolute measure of wealth but rather a measure of the household ownership of selected assets and the availability of basic community-level services (eg, water and electricity).^13^ However, the DHS data were collected from surveys with similar designs and a unified wealth index that facilitated the system used to measure and compare anemia inequalities across the countries and over time.^20^ Another limitation was that the DHS data used from the different countries often were obtained from different years and periods between surveys. Therefore, caution should be exercised when making comparisons of anemia inequalities across countries and times.^21^

## Conclusions

Despite the nearly 60% of exhibited decreases among LMICs, most of these countries continue to face the challenge of a high prevalence of anemia among children and nonpregnant women. Moreover, anemia inequalities among children and nonpregnant women persisted in most LMICs. More effective interventions designed to alleviate the anemia burden among children and nonpregnant women are needed that devote greater attention to socioeconomically disadvantaged populations.

## Notes

Supplement. **eMethods 1.** Study Population and Sample Size **eMethods 2.** Estimation of Anemia Inequalities for Another Dimension of Socioeconomic Position: Education (or Maternal Education in the Case of Children) **eFigure 1.** Flow Diagram of Sample Selection From DHS Before 2014 **eFigure 2.** Prevalence of Total and Severe Anemia Among Children in Low- and Middle-Income Countries (LMICs) **eFigure 3.** Prevalence of Total and Severe Anemia Among Nonpregnant Women in Low- and Middle-Income Countries (LMICs) **eFigure 4.** Annualized Absolute Change in the Prevalence of Total and Severe Anemia in Children in Low- and Middle-Income Countries (LMICs) **eFigure 5.** Annualized Absolute Change in the Prevalence of Total and Severe Anemia in Nonpregnant Women in Low- and Middle-Income Countries (LMICs) **eTable 1.** Sample Sizes and the Prevalence of Total Anemia and Severe Anemia Among Children for Each Country at the Time of the Most Recent Survey **eTable 2.** Sample Sizes and the Prevalence of Total Anemia and Severe Anemia Among Nonpregnant Women at the Time of the Most Recent Survey for Each Country **eTable 3.** Sample Sizes and the Annualized Changes of Total Anemia and Severe Anemia Prevalence Among Children Between the Earliest and Most Recent Survey for Each Country **eTable 4.** Sample Sizes and the Annualized Changes of Total Anemia and Severe Anemia Prevalence Among Nonpregnant Women Between the Earliest and Most Recent Surveys for Each Country **eTable 5.** The Absolute and Relative Inequality of Total Anemia Among Children for Each Country at the Most Recent Survey **eTable 6.** The Absolute and Relative Inequality of Total Anemia Among Nonpregnant Women for Each Country at the Most Recent Survey **eTable 7.** The Annualized Changes of the Absolute and Relative Inequality of Total Anemia Among Children Between the Earliest and Most Recent Survey for Each Country **eTable 8.** The Annualized Changes of the Absolute and Relative Inequality of Total Anemia Among Nonpregnant Women Between the Earliest and Most Recent Survey for Each Country **eTable 9.** Education-Related Absolute and Relative Inequality of Total Anemia Among Children for Each Country at the Most Recent Survey **eTable 10.** Education-Related Absolute and Relative Inequality of Total Anemia Among Nonpregnant Women for Each Country at the Most Recent Survey **eTable 11.** The Annualized Changes of Education-Related Absolute and Relative Inequality of Total Anemia Among Children Between the Earliest and Most Recent Survey for Each Country **eTable 12.** The Annualized Changes of Education-Related Absolute and Relative Inequality of Total Anemia Among Nonpregnant Women Between the Earliest and Most Recent Survey for Each Country
