# Supplementary material for: Trends in Socioeconomic Inequalities and Prevalence of Anemia Among Children and Nonpregnant Women in Low- and Middle-Income Countries
Source: JAMA Netw Open. 2018 Sep 28;1(5):e182899. doi: 10.1001/jamanetworkopen.2018.2899 (PMC6324516; doi:10.1001/jamanetworkopen.2018.2899)
Supplement: Supplement. — eMethods 1. Study Population and Sample Size eMethods 2. Estimation of Anemia Inequalities for Another Dimension of Socioeconomic Position: Education (or Maternal Education in the Case of Children) eFigure 1. Flow Diagram of Sample Selection From DHS Before 2014 eFigure 2. Prevalence of Total and Severe Anemia Among Children in Low- and Middle-Income Countries (LMICs) eFigure 3. Prevalence of Total and Severe Anemia Among Nonpregnant Women in Low- and Middle-Income Countries (LMICs) eFigure 4. Annualized Absolute Change in the Prevalence of Total and Severe Anemia in Children in Low- and Middle-Income Countries (LMICs) eFigure 5. Annualized Absolute Change in the Prevalence of Total and Severe Anemia in Nonpregnant Women in Low- and Middle-Income Countries (LMICs) eTable 1. Sample Sizes and the Prevalence of Total Anemia and Severe Anemia Among Children for Each Country at the Time of the Most Recent Survey eTable 2. Sample Sizes and the Prevalence of Total Anemia and Severe Anemia Among Nonpregnant Women at the Time of the Most Recent Survey for Each Country eTable 3. Sample Sizes and the Annualized Changes of Total Anemia and Severe Anemia Prevalence Among Children Between the Earliest and Most Recent Survey for Each Country eTable 4. Sample Sizes and the Annualized Changes of Total Anemia and Severe Anemia Prevalence Among Nonpregnant Women Between the Earliest and Most Recent Surveys for Each Country eTable 5. The Absolute and Relative Inequality of Total Anemia Among Children for Each Country at the Most Recent Survey eTable 6. The Absolute and Relative Inequality of Total Anemia Among Nonpregnant Women for Each Country at the Most Recent Survey eTable 7. The Annualized Changes of the Absolute and Relative Inequality of Total Anemia Among Children Between the Earliest and Most Recent Survey for Each Country eTable 8. The Annualized Changes of the Absolute and Relative Inequality of Total Anemia Among Nonpregnant Women Between the Earliest and Most Recent Survey [file jamanetwopen-1-e182899-s001.pdf]

Supplementary Online Content

Yang F, Liu X, Zha P. Trends in socioeconomic inequalities and prevalence of anemia among children and nonpregnant women in low- and middle-income countries. *JAMA Netw Open.* 2018;1(5):e182899. doi:10.1001/jamanetworkopen.2018.2899

eMethods 1. Study Population and Sample Size
eMethods 2. Estimation of Anemia Inequalities for Another Dimension of Socioeconomic Position: Education (or Maternal Education in the Case of Children)
eFigure 1. Flow Diagram of Sample Selection From DHS Before 2014
eFigure 2. Prevalence of Total and Severe Anemia Among Children in Low- and Middle-Income Countries (LMICs)
eFigure 3. Prevalence of Total and Severe Anemia Among Nonpregnant Women in Low- and Middle-Income Countries (LMICs)
eFigure 4. Annualized Absolute Change in the Prevalence of Total and Severe Anemia in Children in Low- and Middle-Income Countries (LMICs)
eFigure 5. Annualized Absolute Change in the Prevalence of Total and Severe Anemia in Women in Low- and Middle-Income Countries (LMICs)
eTable 1. Sample Sizes and the Prevalence of Total Anemia and Severe Anemia Among Children for Each Country at the Time of the Most Recent Survey
eTable 2. Sample Sizes and the Prevalence of Total Anemia and Severe Anaemia Among Nonpregnant Women at the Time of the Most Recent Survey for Each Country
eTable 3. Sample Sizes and the Annualized Changes of Total Anemia and Severe Anemia Prevalence Among Children Between the Earliest and Most Recent Survey for Each Country
eTable 4. Sample Sizes and the Annualized Changes of Total Anemia and Severe Anemia Prevalence Among Nonpregnant Women Between the First and Most Recent Surveys for Each Country
eTable 5. The Absolute and Relative Inequality of Total Anemia Among Children for Each Country at the Most Recent Survey
eTable 6. The Absolute and Relative Inequality of Total Anemia Among Nonpregnant Women for Each Country at the Most Recent Survey
eTable 7. The Annualized Changes of the Absolute and Relative Inequality of Total Anemia Among Children Between the Earliest and Most Recent Survey for Each Country
eTable 8. The Annualized Changes of the Absolute and Relative Inequality of Total Anemia Among Nonpregnant Women Between the Earliest and Most Recent Survey for Each Country
eTable 9. Education-Related Absolute and Relative Inequality of Total Anemia Among Children for Each Country at the Most Recent Survey
eTable 10. Education-Related Absolute and Relative Inequality of Total Anemia Among Nonpregnant Women for Each Country at the Most Recent Survey
eTable 11. The Annualized Changes of Education-Related Absolute and Relative Inequality of Total Anemia Among Children Between the Earliest and Most Recent Survey for Each Country
eTable 12. The Annualized Changes of Education-Related Absolute and Relative Inequality of Total Anemia Among Nonpregnant Women Between the Earliest and Most Recent Survey for Each Country

This supplementary material has been provided by the authors to give readers additional information about their work.

© 2018 Yang F et al. *JAMA Network Open*.

eMethods 1. Study Population and Sample Size

The DHS are large representative household surveys conducted in 85 LMICs; 57 of these countries conducted at least 2 surveys. These multiple surveys allow for repeated cross-sectional analyses that can evaluate trends in the socioeconomic inequalities and prevalence of anemia. We constructed cross-sectional and repeated cross-sectional datasets using women's and/or children's surveys from DHS surveys between January 1, 2000, and December 31, 2014 (eFigure 1). The DHS only collect information on hemoglobin concentrations of either women only or children only in some countries. For example, surveys conducted in the Congo determined the hemoglobin concentrations among women in 2005 and 2011, but did so only once for children. Given this limitation, we constructed separate cross-sectional and repeated cross-sectional datasets for women and children. In addition, the DHS population coverage was generally children aged 0–59 months, women aged 15–49 years. Some countries have extended the age range of the women surveyed, for example, 13–49 years or 15–64 years, as well as a small sample of pregnant women in the DHS of each country. The DHS did not collect information about hemoglobin concentrations for children aged 0–5 months. Therefore, all analysis was limited to non-pregnant women aged 15–49 years and children aged 6–59 months.

Of the 85 LMICs, the surveys for 40 of them had no hemoglobin concentration data. The remaining 45 LMICs were used to construct the cross-sectional and repeated cross-sectional datasets. The first and second datasets were developed from cross-sectional surveys that included the countries where at least one survey had been conducted. Data from the most recent surveys that captured hemoglobin concentrations and socio-demographic characteristics for children aged 6–59 months and non-pregnant women aged 15–49 years were selected to

© 2018 Yang F et al. *JAMA Network Open*.

construct the dataset. Therefore, we included the most recent surveys of the 45 LMICs with hemoglobin concentration data in the datasets. The first was a cross-sectional dataset of children aged 6–59 months developed from the most recent surveys of these 45 countries, and, after excluding 133,067 children aged < 6 months and/or those with missing data, were left with the final analytic dataset containing 163,419 children aged 6–59 months (eFigure 1). Of the most recent of the surveys, conducted from 2005 through 2014 for these countries, 13 were conducted between 2005 and 2009, 14 in 2010 or 2011, and 18 between 2012 and 2014 (eTable 1). The second survey was a cross-sectional dataset of non-pregnant women aged 15–49 years developed from the most recent surveys of these 45 countries, and, after excluding 278,306 women aged < 15 or > 49 years and/or currently pregnant and/or those with missing data, were left with the final analytic dataset containing 304,202 non-pregnant women aged 15–49 years (eFigure 1). The most recent of the surveys, conducted from 2005 through 2014, for these countries included 12 surveys conducted between 2006 and 2009, 16 in 2010 or 2011, and 17 between 2012 and 2014 (eTable 2).

The third and fourth datasets were developed from repeated cross-sectional surveys across countries in which more than 2 surveys were completed and had captured hemoglobin concentrations and socio-demographic characteristics of children aged 6–59 months and non-pregnant women aged 15–49 years. If a country had at least 2 DHS surveys available, the data of the earliest and most recent surveys were selected to construct the datasets. Therefore, we included the earliest and the most recent surveys of the 24 LMICs in the third dataset for children aged 6–59 months. Of the remaining 61 LMICs, 40 had no hemoglobin concentration data and 21 had only one survey that captured hemoglobin concentrations. The third dataset consisted of

© 2018 Yang F et al. *JAMA Network Open*.

information concerning children aged 6–59 months developed from both the earliest and the most recent surveys of 24 LMICs. After excluding 133,067 children aged < 6 months and/or those with missing data, we were left with dataset containing 182,273 children aged 6–59 months (eFigure 1). The average time between the earliest and the most recent surveys for children was 7.4 years (standard deviation, 2.5 years), with a minimum of 5 years in Armenia (2000–2005), Bolivia (2003–2008), Jordan (2007–2012), Lesotho (2004–2009), Madagascar (2003–2008), Nepal (2006–2011), Sierra Leone (2008–2013), and Zimbabwe (2005–2010), and a maximum of 12 years in Haiti (2000–2012) and Peru (2000–2012) (eTable 3). We included the earliest and the most recent surveys of 25 LMICs in the fourth dataset. Of the remaining 60 LMICs, 40 had no hemoglobin concentration data and 20 had only 1 survey that captured hemoglobin concentrations. After excluding 352,284 women aged < 15 or > 49 years and/or currently pregnant and/or those with missing data, the fourth repeated cross-sectional surveys included a total of 322,088 non-pregnant women from the earliest and the most recent surveys from these 25 LMICs (eFigure 1). The average time between the earliest and the most recent surveys was 7.2 years (standard deviation, 2.4 years), with a minimum of 5 years in Armenia (2000–2005), Bolivia (2003–2008), Jordan (2007–2012), Lesotho (2004–2009), Madagascar (2003–2008), Nepal (2006–2011), Senegal (2005–2010), Sierra Leone (2008–2013), and Zimbabwe (2005–2010), and a maximum of 12 years in Haiti (2000–2012) and Peru (2000–2012) (eTable 4).

© 2018 Yang F et al. *JAMA Network Open*.

eMethods 2. Estimation of Anemia Inequalities for Another Dimension of Socioeconomic Position: Education (or Maternal Education in the Case of Children)

We also examined anemia inequalities for another dimension of socioeconomic position: education (or maternal education in the case of children) using the same analysis method as that employed when examining household wealth. Education was measured as the reported number of education years. The children and non-pregnant women in this study were separately ranked from the lowest education level (rank 0) to the highest (rank 1) in order to estimate their positions in the cumulative distribution of socioeconomic status.[15] The education-related slope index of inequality (SII) and the relative index of inequality (RII) were calculated to determine absolute and relative socioeconomic inequalities of anemia, respectively. The annualized changes of the education-related SII and RII were calculated to adjust for the time difference between the earliest and the most recent surveys.

The education-related SII and RII of total anaemia among children was −8.37% and 0.71 (eTable 9). For children, the education-related SII and RII were significantly negative and less than 1 in 32 of 45LMICs, respectively (eTable 9). The education-related SII and RII of total anaemia among non-pregnant women was −4.88% and 0.80, respectively (eTable 10). For non-pregnant women, the education-related SII and RII were significantly negative and less than 1 in 25 countries, respectively (eTable 10). For example, the education-related SII for Ethiopia was -16.79 (-18.94 to -14.63), signifying that moving from the bottom to the top of the education distribution was associated with an estimated decrease of 16.79 cases of anemia per 100 non-pregnant women (eTable 10). Among children, the annualized changes in the education-related SII were not significantly different from 0 in 18 of the 24 LMICs (eTable 11). Among pregnant women, the

© 2018 Yang F et al. *JAMA Network Open*.

changes in the education-related SII were not significantly different from 0 in 15 of the 25 LMICs. However, the changes in the education-related SII for the countries of Sierra Leone, Guinea, and Cameroon represented annualized decreases among non-pregnant women, indicating an increase in socioeconomic inequalities (eTable 12).

© 2018 Yang F et al. *JAMA Network Open*.

eFigure 1. Flow Diagram of Sample Selection From DHS Before 2014

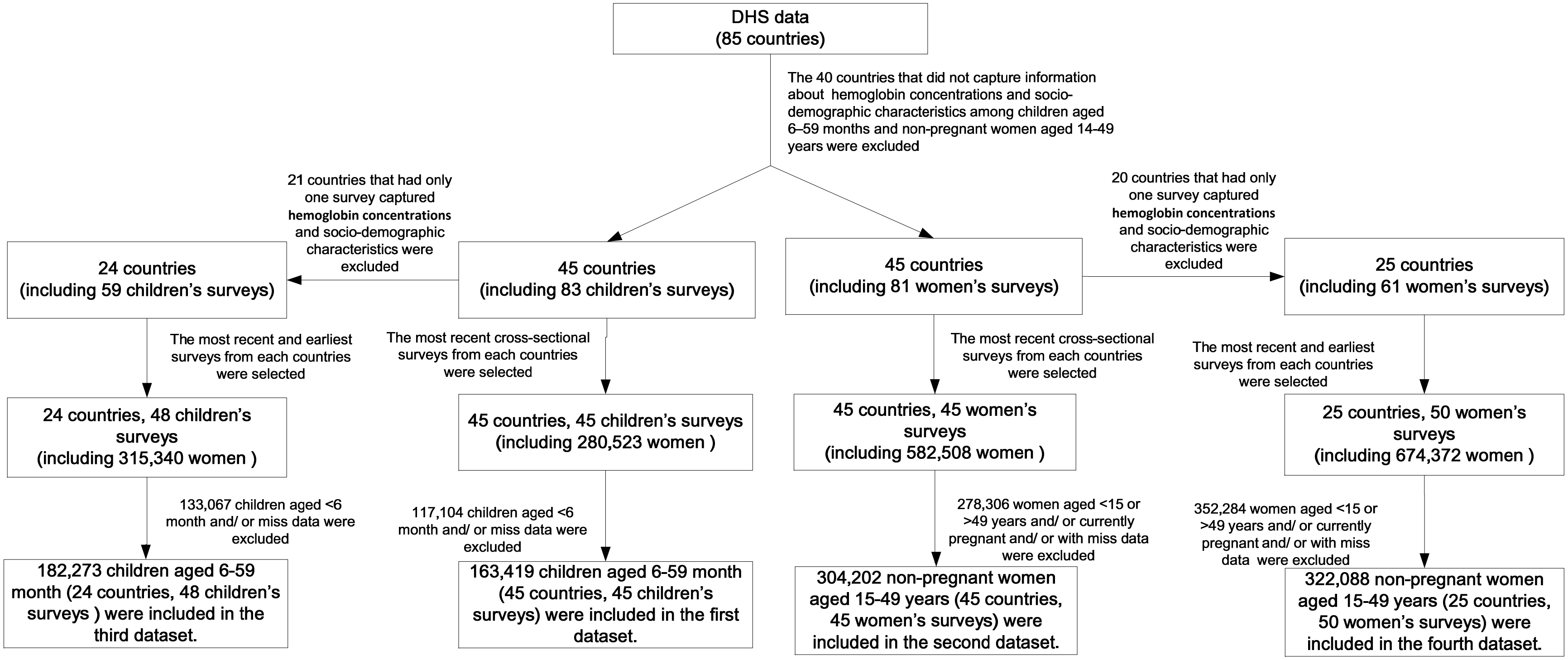

© 2018 Yang F et al. *JAMA Network Open*.

eFigure 2. Prevalence of Total and Severe Anemia Among Children in Low- and Middle-Income Countries (LMICs)

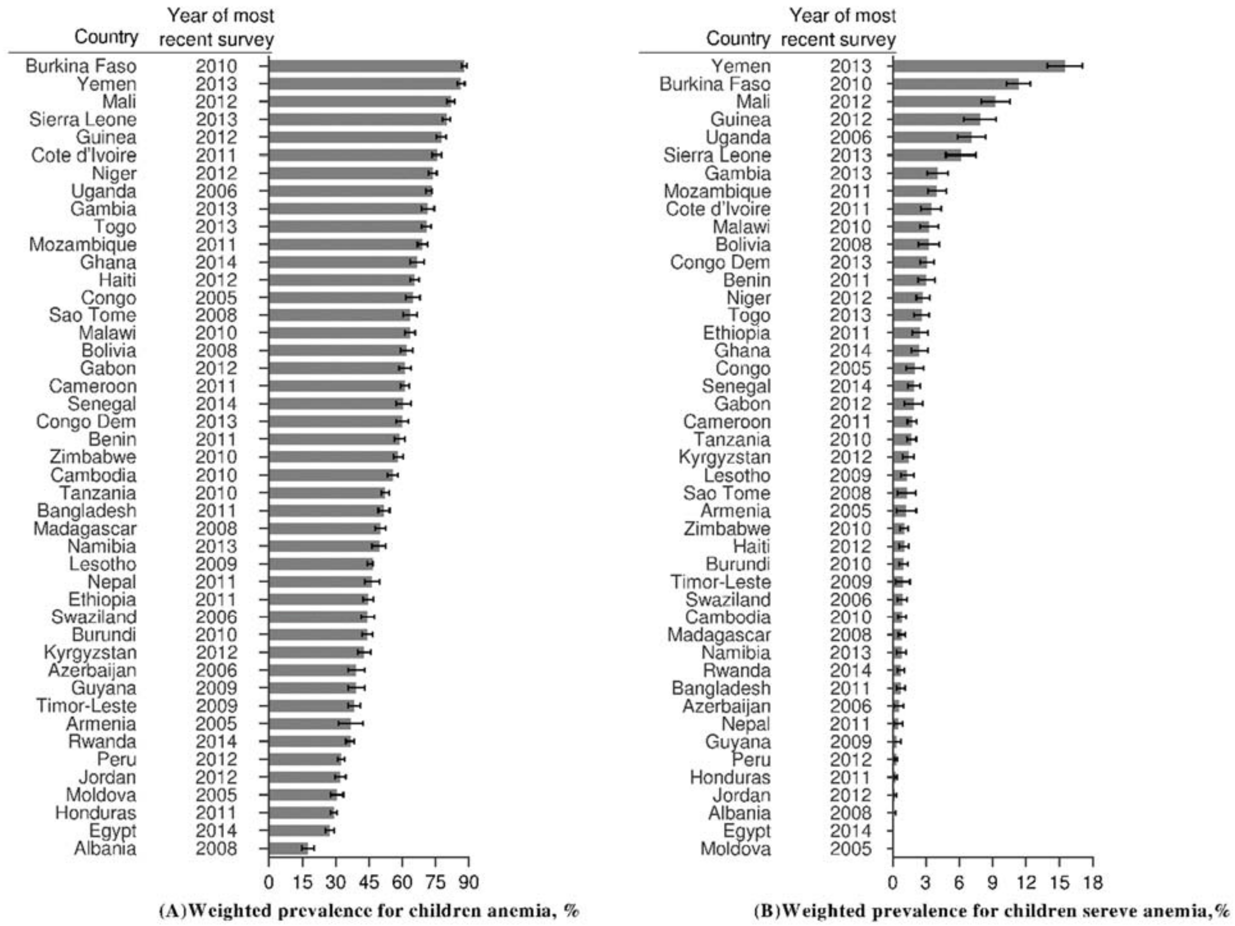

Weighted prevalence of total and severe anemia among children aged 6 to 59 months are shown for each LMIC. Error bars represent 95% CIs. Specific numbers of participants, prevalence total and severe anemia, and 95% CIs are provided in eTables 1 and 2 and omitted here for clarity.

© 2018 Yang F et al. *JAMA Network Open*.

eFigure 3. Prevalence of Total and Severe Anemia Among Nonpregnant Women in Low- and Middle-Income Countries (LMICs)

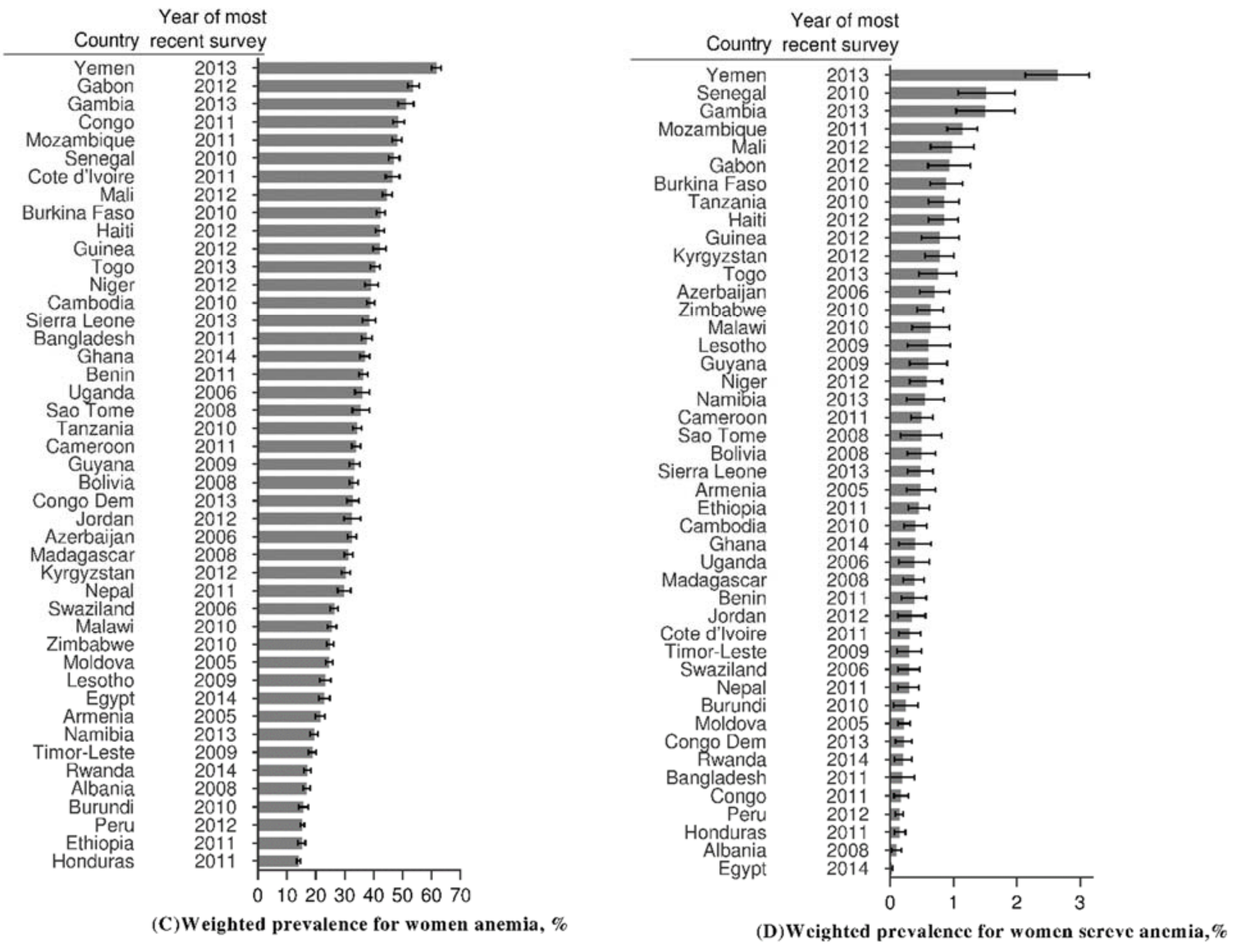

Weighted prevalence of total and severe anemia among nonpregnant women aged 15 to 49 years are shown for each LMIC. Error bars represent 95% CIs. Specific numbers of participants, prevalence total and severe anemia, and 95% CIs are provided in eTables 1 and 2 and omitted here for clarity.

© 2018 Yang F et al. *JAMA Network Open*.

eFigure 4. Annualized Absolute Change in the Prevalence of Total and Severe Anemia in Children in Low- and Middle-Income Countries (LMICs)

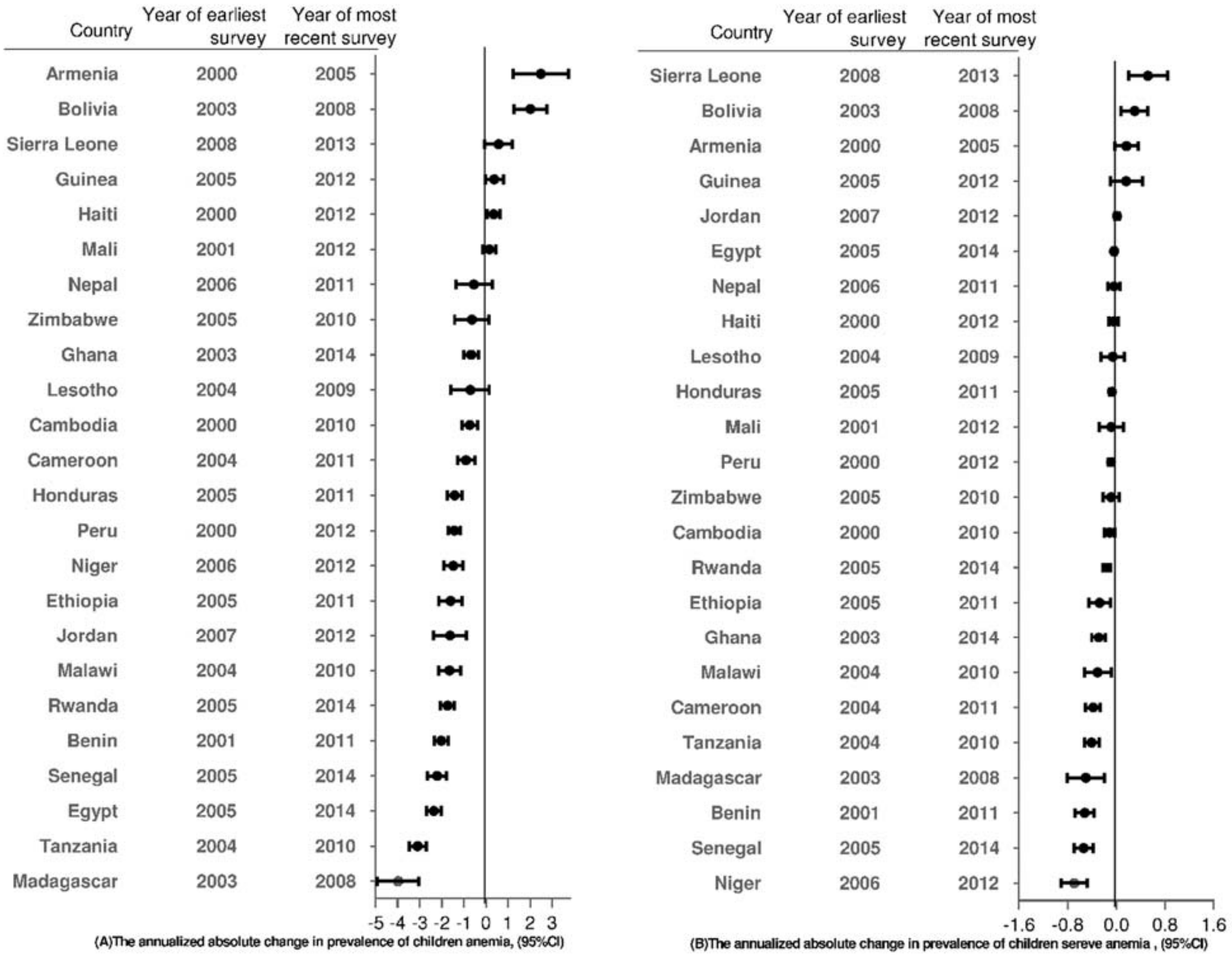

Annualized absolute changes in prevalence of total and severe anemia among children aged 6 to 59 months are shown for each LMIC. Error bars represent 95% CI. Specific numbers of participants, annualized absolute changes in the prevalence of total and severe anemia, and 95% CIs are provided in eTables 3 and 4 and omitted here for clarity.

© 2018 Yang F et al. *JAMA Network Open*.

eFigure 5. Annualized Absolute Change in the Prevalence of Total and Severe Anemia in Women in Low- and Middle-Income Countries (LMICs)

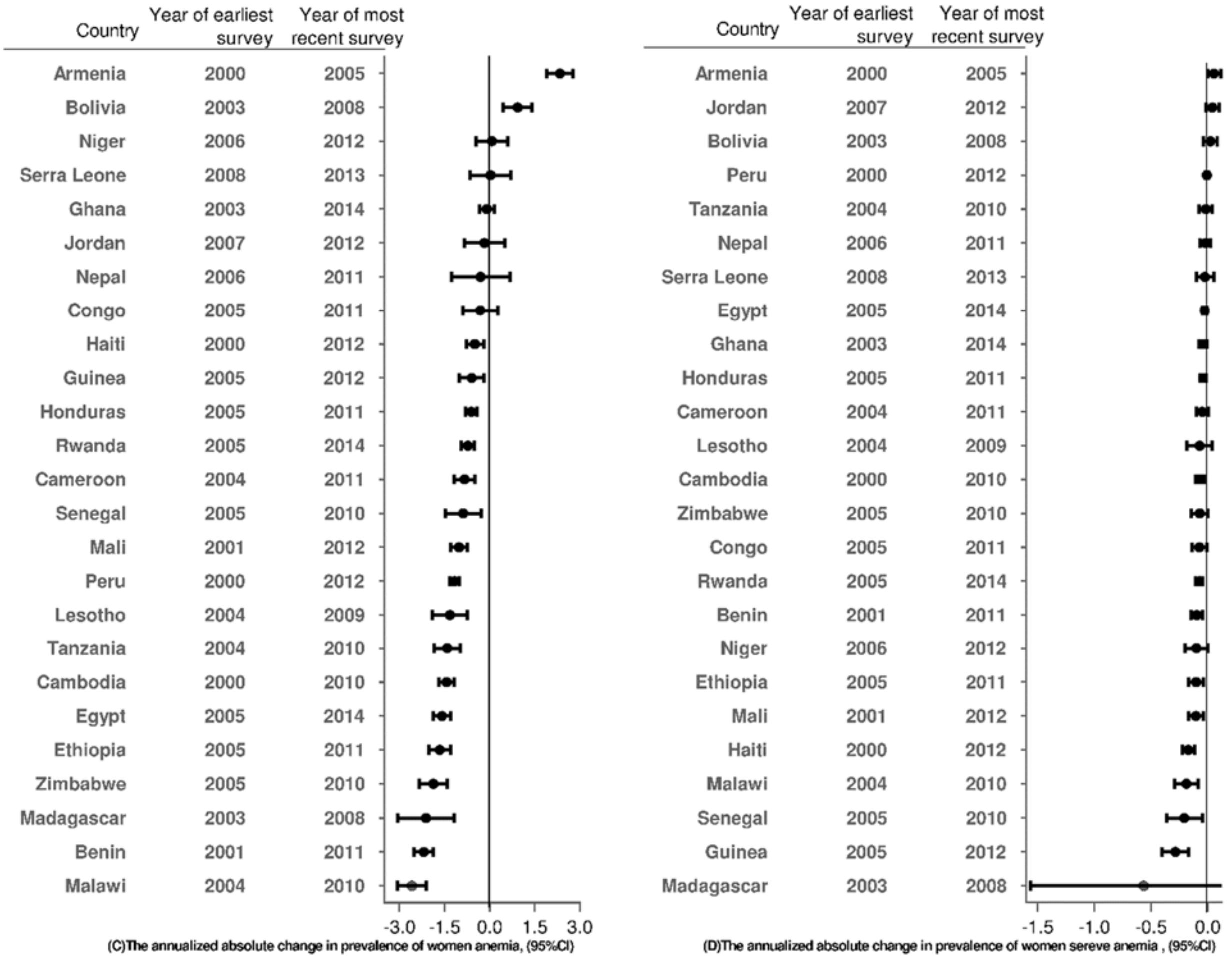

Annualized absolute changes in prevalence of total and severe anemia among nonpregnant women aged 15 to 49 years are shown for each LMIC. Error bars represent 95% CI. Specific numbers of participants, annualized absolute changes in the prevalence of total and severe anemia, and 95% CIs are provided in eTables 3 and 4 and omitted here for clarity.

© 2018 Yang F et al. *JAMA Network Open*.

eTable 1. Sample Sizes and the Prevalence of Total Anemia and Severe Anemia Among Children for Each Country at the Time of the Most Recent Survey

Country	Survey year	Sample sizes (N)	Total anaemia				Severe anaemia			
			n	Prevalence (%)	95% Confidence Interval		n	Prevalence (%)	95%Confidence Interval	
Albania	2008	1,355	254	17.57	14.81	20.32	2	0.11	-0.08	0.30
Armenia	2005	1,024	332	36.82	31.39	42.25	15	1.24	0.34	2.13
Azerbaijan	2006	1,812	671	39.35	35.49	43.20	9	0.55	0.10	0.99
Bangladesh	2011	2,283	1,191	51.77	49.22	54.32	18	0.72	0.34	1.10
Benin	2011	3,312	1,944	58.87	56.59	61.15	96	3.04	2.28	3.79
Bolivia	2008	2,378	1,445	61.98	59.17	64.78	69	3.27	2.31	4.24
Burkina Faso	2010	5,928	5,239	87.89	86.73	89.04	647	11.32	10.23	12.41
Burundi	2010	3,080	1,327	44.29	42.02	46.56	25	0.97	0.58	1.37
Gabon	2012	3,049	1,943	61.28	58.61	63.95	54	1.92	1.09	2.74
Gambia	2013	2,931	2,167	71.52	68.64	74.40	137	4.05	3.14	4.97
Cambodia	2010	3,394	1,840	55.82	53.49	58.15	27	0.85	0.48	1.22
Cameroon	2011	4,566	2,835	61.26	59.30	63.22	93	1.74	1.34	2.15
Congo Dem	2013	7,317	4,613	60.07	57.40	62.74	274	3.07	2.46	3.69
Congo	2005	1,976	1,292	64.84	61.55	68.13	36	1.99	1.23	2.76
Cote d'Ivoire	2011	2,805	2,113	75.69	73.58	77.80	99	3.47	2.56	4.39
Egypt	2014	4,603	1,317	27.42	25.46	29.37	1	0.01	-0.01	0.02
Ethiopia	2011	8,510	4,277	44.60	42.24	46.96	306	2.45	1.74	3.16
Ghana	2014	2,388	1,662	66.80	63.63	69.97	67	2.41	1.66	3.17
Guinea	2012	2,793	2,128	77.58	75.26	79.90	211	7.87	6.42	9.31
Guyana	2009	1,427	559	39.29	35.50	43.07	8	0.38	0.03	0.74
Haiti	2012	3,509	2,297	65.64	63.58	67.70	33	1.02	0.59	1.45
Honduras	2011	8,538	2,592	29.30	27.92	30.67	21	0.25	0.12	0.39
Jordan	2012	5,522	1,755	32.21	29.81	34.60	18	0.22	0.09	0.36
Kyrgyzstan	2012	3,617	1,592	42.93	40.03	45.83	54	1.42	0.90	1.94
Lesotho	2009	2,084	1,025	46.84	44.23	45.82	31	1.30	0.75	1.94
Madagascar	2008	4,750	2,418	50.15	47.89	52.42	43	0.81	0.50	1.13
Malawi	2010	4,177	2,683	63.57	61.33	65.81	127	3.29	2.46	4.11
Mali	2012	4,196	3,401	81.92	80.13	83.71	361	9.26	7.99	10.53
Moldova	2005	1,261	369	30.65	27.76	33.54	0	0.00	0.00	0.00
Mozambique	2011	4,361	2,889	69.18	66.95	71.41	147	3.99	3.17	4.81
Namibia	2013	1,537	785	49.58	46.48	52.68	15	0.80	0.36	1.23
Nepal	2011	2,088	961	46.40	42.98	49.83	10	0.51	0.09	0.94
Niger	2012	4,384	3,283	73.71	71.84	75.59	123	2.73	2.13	3.32
Peru	2012	8,987	3,192	32.57	31.08	34.09	31	0.32	0.19	0.45
Rwanda	2014	3,230	1,158	36.62	34.64	38.60	24	0.73	0.42	1.03
Sao Tome	2008	1,540	930	63.61	60.38	66.84	16	1.25	0.44	2.06

© 2018 Yang F et al. *JAMA Network Open*.

Senegal	2014	5,421	3,318	60.52	57.21	63.83	120	1.94	1.38	2.51
Sierra Leone	2013	4,140	3,329	79.95	78.12	81.78	274	6.15	4.79	7.50
Swaziland	2006	1,785	783	44.44	41.53	47.35	17	0.87	0.43	1.31
Tanzania	2010	6,851	3,673	52.35	50.49	54.21	106	1.72	1.32	2.12
Timor-Leste	2009	2,377	988	38.45	35.62	41.27	22	0.93	0.30	1.57
Togo	2013	2,890	2,052	70.95	68.77	73.13	76	2.62	1.94	3.30
Uganda	2006	2,110	1,551	73.23	70.66	73.12	142	7.14	5.89	8.39
Yemen	2013	3,754	3,316	86.56	84.95	88.17	644	15.49	13.91	17.08
Zimbabwe	2010	3,379	1,973	58.16	56.02	60.29	42	1.04	0.68	1.40

According to WHO standards, total anaemia was defined as Hb < 11 g/dL for children aged 6-59 months; severe anaemia was defined as Hb < 7 g/dL.[9,17] We used the above cross-sectional data set (the first data set) for the estimates of anaemia prevalence among children. All estimates used to determine anemia were weighted and proved appropriate for the DHS complex survey design

© 2018 Yang F et al. *JAMA Network Open*.

eTable 2. Sample Sizes and the Prevalence of Total Anemia and Severe Anaemia Among Nonpregnant Women at the Time of the Most Recent Survey for Each Country

Country	Survey year	Sample sizes (N)	Total anaemia				Severe anaemia			
			n	Prevalence (%)	95%Confidence Interval		n	Prevalence (%)	95%Confidence Interval	
Albania	2008	7,333	1,312	16.94	15.72	18.16	7	0.10	0.02	0.18
Armenia	2005	5,957	1,394	21.56	19.79	23.33	34	0.48	0.26	0.71
Azerbaijan	2006	7,835	2,835	32.58	31.04	34.12	66	0.70	0.47	0.94
Bangladesh	2011	5,314	2,194	37.66	35.90	39.42	8	0.19	0.00	0.38
Benin	2011	4,579	1,863	36.45	34.87	38.03	21	0.38	0.18	0.57
Bolivia	2008	5,400	1,988	33.16	31.63	34.69	31	0.49	0.27	0.71
Burkina Faso	2010	7,556	3,569	42.53	41.03	44.03	71	0.89	0.63	1.14
Burundi	2010	4,034	670	15.75	13.99	17.51	8	0.25	0.05	0.44
Cambodia	2010	8,754	3,796	39.05	37.73	40.37	32	0.40	0.22	0.58
Cameroon	2011	7,067	2,748	33.90	32.37	35.43	43	0.50	0.33	0.67
Congo	2011	4,980	2,604	48.61	46.60	50.62	13	0.17	0.06	0.29
Congo Dem	2013	8,125	3,212	32.89	30.77	35.01	24	0.22	0.09	0.34
Cote d'Ivoire	2011	4,197	2,175	46.56	44.16	48.96	21	0.31	0.14	0.48
Egypt	2014	6,464	1,573	22.99	21.13	24.85	3	0.02	0.00	0.04
Ethiopia	2011	14,342	2,750	15.14	13.93	16.35	111	0.46	0.29	0.62
Gabon	2012	4,907	2,859	53.77	51.74	55.80	46	0.93	0.60	1.27
Gambia	2013	4,182	2,551	51.13	48.44	53.82	75	1.50	1.04	1.97
Ghana	2014	4,352	1,803	36.92	35.13	38.71	14	0.39	0.14	0.65
Guinea	2012	4,203	1,983	42.08	39.94	44.22	35	0.79	0.50	1.09
Guyana	2009	4,388	1,587	33.34	31.55	35.13	24	0.60	0.31	0.90
Haiti	2012	8,817	4,228	42.18	40.59	43.77	89	0.85	0.61	1.08
Honduras	2011	20,385	3,112	14.08	13.41	14.75	23	0.15	0.07	0.24
Jordan	2012	6,247	2,385	32.60	29.72	35.48	20	0.34	0.13	0.56
Kyrgyzstan	2012	7,469	2,566	30.47	28.92	32.02	68	0.78	0.55	1.01
Lesotho	2009	3,713	973	23.31	21.45	25.17	21	0.61	0.28	0.95
Madagascar	2008	7,583	2,726	31.36	29.92	32.80	32	0.38	0.21	0.54
Malawi	2010	6,601	1,869	25.69	24.17	27.21	36	0.64	0.35	0.94
Mali	2012	4,565	2,313	44.64	42.94	46.34	52	0.98	0.64	1.32
Moldova	2005	6,932	1,871	24.61	23.36	25.86	18	0.22	0.12	0.32
Mozambique	2011	12,137	6,369	48.06	46.43	49.69	152	1.14	0.90	1.38
Namibia	2013	4,053	834	19.38	17.96	20.80	17	0.55	0.26	0.85
Nepal	2011	5,794	1,837	29.84	27.58	32.10	17	0.30	0.13	0.46
Niger	2012	4,369	1,848	39.24	36.95	41.53	33	0.57	0.31	0.82
Peru	2012	22,514	4,207	15.41	14.75	16.07	35	0.15	0.08	0.21
Rwanda	2014	6,212	1,175	17.13	15.90	18.36	13	0.20	0.07	0.34
Sao Tome	2008	2,318	967	35.61	32.64	38.58	11	0.49	0.17	0.81

© 2018 Yang F et al. *JAMA Network Open*.

Senegal	2010	5,085	2,709	47.11	45.23	48.99	85	1.52	1.08	1.97
Sierra Leone	2013	7,181	3,206	38.44	36.14	40.74	33	0.48	0.28	0.68
Swaziland	2006	4,343	1,296	26.43	25.08	27.78	17	0.30	0.12	0.47
Tanzania	2010	8,990	3,794	34.33	32.86	35.80	103	0.85	0.61	1.09
Timor-Leste	2009	3,803	825	18.82	17.38	20.26	12	0.30	0.11	0.49
Togo	2013	4,374	1,945	40.58	38.91	42.25	27	0.76	0.46	1.05
Uganda	2006	2,477	979	36.05	33.57	38.53	11	0.38	0.14	0.62
Yemen	2013	6,760	4,992	61.70	60.03	63.37	204	2.64	2.13	3.14
Zimbabwe	2010	7,511	2,194	24.99	23.70	26.28	55	0.64	0.43	0.84

According to WHO standards, total anaemia was defined as Hb < 12 g/dL for non-pregnant women aged 15–49 years; severe anaemia was defined as Hb < 8 g/dL. We used the above cross-sectional data set (the second data set) for the estimates of anaemia prevalence among non-pregnant women. All estimates used to determine anemia were weighted and proved appropriate for the DHS complex survey design. Estimates of the prevalence of total and severe anemia among non-pregnant women were adjusted using world population data obtained from the WHO.

© 2018 Yang F et al. *JAMA Network Open*.

eTable 3. Sample Sizes and the Annualized Changes of Total Anemia and Severe Anemia Prevalence Among Children Between the Earliest and Most Recent Survey for Each Country

Country	The earliest survey		The most recent survey		Total anaemia (percentage points)			Severe anaemia (percentage points)		
	Survey year	Sample sizes (N)	Survey year	Sample sizes (N)	The annualized change	95%Confidence Interval		The annualized change	95%Confidence Interval	
Armenia	2000	1518	2005	1024	2.48	1.22	3.74	0.170	-0.016	0.357
Benin	2001	2316	2011	3312	−2.03	−2.34	−1.71	−0.516	−0.672	−0.359
Bolivia	2003	2873	2008	2378	2.02	1.27	2.77	0.304	0.086	0.523
Cambodia	2000	1740	2010	3394	−0.73	−1.09	−0.38	−0.105	−0.188	−0.022
Cameroon	2004	3292	2011	4566	−0.90	−1.29	−0.51	−0.379	−0.498	−0.259
Egypt	2005	3853	2014	4603	−2.36	−2.69	−2.03	−0.032	−0.054	−0.010
Ethiopia	2005	3394	2011	8510	−1.61	−2.12	−1.09	−0.272	−0.446	−0.098
Ghana	2003	3183	2014	2388	−0.67	−0.99	−0.34	−0.289	−0.399	−0.179
Guinea	2005	2686	2012	2793	0.38	−0.03	0.78	0.166	−0.098	0.430
Haiti	2000	2836	2012	3509	0.35	0.07	0.63	−0.038	−0.115	0.039
Honduras	2005	8258	2011	8538	-1.41	-1.74	-1.08	-0.072	-0.111	-0.033
Jordan	2007	4124	2012	5522	-1.63	-2.39	-0.88	0.015	-0.023	0.053
Lesotho	2004	1322	2009	2084	-0.71	-1.57	0.15	-0.056	-0.247	0.135
Madagascar	2003	1542	2008	4750	−3.98	−4.91	−3.04	−0.499	−0.805	−0.192
Malawi	2004	2329	2010	4177	−1.65	−2.15	−1.14	−0.305	−0.527	−0.083
Mali	2001	2961	2012	4196	0.16	−0.14	0.46	−0.081	−0.276	0.114
Nepal	2006	4693	2011	2088	−0.55	−1.37	0.27	−0.033	−0.135	0.069
Niger	2006	3612	2012	4384	−1.48	−1.90	−1.05	−0.689	−0.903	−0.474
Peru	2000	2519	2012	8987	-1.43	-1.69	-1.17	-0.084	-0.127	-0.042
Rwanda	2005	3747	2014	3230	−1.74	−2.06	−1.43	−0.151	−0.213	−0.088
Senegal	2005	2733	2014	5421	−2.21	−2.64	−1.78	−0.537	−0.690	−0.384
Sierra Leone	2008	1894	2013	4140	0.57	−0.06	1.20	0.521	0.201	0.841
Tanzania	2004	7230	2010	6851	-3.09	-3.48	-2.71	-0.404	-0.529	-0.280
Zimbabwe	2005	3394	2010	3379	−0.64	−1.41	0.14	−0.085	−0.216	0.046

According to WHO standards, total anaemia was defined as Hb < 11 g/dL for children aged 6-59 months; severe anaemia was defined as Hb < 7 g/dL.[9,17] We used above repeated cross-sectional dataset (The third dataset) for the analyses of the annualized changes. All estimates used to determine anemia were weighted and proved appropriate for the DHS complex survey design. The changes in the prevalences of total and severe anemia were measured by the annual absolute change in percentage points and were estimated through a calculation of the difference between anemia prevalence in the earliest and most recent surveys divided by the number of years between these two surveys.

© 2018 Yang F et al. *JAMA Network Open*.

eTable 4. Sample Sizes and the Annualized Changes of Total Anemia and Severe Anemia Prevalence Among Nonpregnant Women Between the First and Most Recent Surveys for Each Country

Country	The earliest survey		The most recent survey		Total anaemia (percentage points)			Severe anaemia (percentage points)		
	Survey year	Sample sizes (n)	Survey year	Sample sizes (n)	The annualized change	95%Confidence Interval		The annualized change	95%Confidence Interval	
Armenia	2000	5953	2005	5957	2.34	1.91	2.78	0.057	-0.001	0.114
Benin	2001	2752	2011	4579	−2.19	−2.50	−1.88	−0.097	−0.150	−0.044
Bolivia	2003	5577	2008	5400	0.93	0.45	1.42	0.024	−0.035	0.083
Cambodia	2000	3397	2010	8754	−1.43	−1.68	−1.18	−0.071	−0.111	−0.031
Cameroon	2004	4605	2011	7067	−0.83	−1.17	−0.49	−0.047	−0.099	0.005
Congo	2005	2943	2011	4980	−0.31	−0.88	0.27	−0.072	−0.138	−0.007
Egypt	2005	5705	2014	6464	−1.58	−1.87	−1.29	−0.027	−0.048	−0.007
Ethiopia	2005	5489	2011	14342	−1.66	−2.03	−1.29	−0.103	−0.169	−0.037
Ghana	2003	4862	2014	4352	−0.09	−0.34	0.15	−0.039	−0.073	−0.005
Guinea	2005	3499	2012	4203	−0.60	−1.02	−0.18	−0.285	−0.404	−0.166
Haiti	2000	4379	2012	8817	−0.49	−0.78	−0.20	−0.170	−0.226	−0.115
Honduras	2005	17343	2011	20385	-0.600	-0.793	-0.407	-0.042	-0.067	-0.018
Jordan	2007	4463	2012	6247	-0.169	-0.846	0.507	0.041	-0.018	0.100
Lesotho	2004	2859	2009	3713	−1.32	−1.91	−0.73	−0.071	−0.182	0.041
Madagascar	2003	2347	2008	7583	−2.12	−3.06	−1.19	−0.564	−1.561	0.434
Malawi	2004	2383	2010	6601	−2.59	−3.07	−2.11	−0.189	−0.293	−0.084
Mali	2001	3467	2012	4565	−1.01	−1.30	−0.73	−0.104	−0.169	−0.040
Nepal	2006	10041	2011	5794	−0.30	−1.27	0.67	−0.020	−0.066	0.026
Niger	2006	3716	2012	4369	0.08	−0.45	0.61	−0.100	−0.202	0.003
Peru	2000	5907	2012	22514	−1.17	−1.33	−1.02	−0.008	−0.020	0.005
Rwanda	2005	5209	2014	6212	−0.72	−0.94	−0.50	−0.075	−0.105	−0.044
Senegal	2005	3974	2010	5085	−0.88	−1.48	−0.27	−0.206	−0.366	−0.047
Sierra Leone	2008	3098	2013	7181	0.03	−0.64	0.70	−0.023	−0.101	0.054
Tanzania	2004	9072	2010	8990	−1.41	−1.83	−0.98	−0.016	−0.074	0.041
Zimbabwe	2005	7383	2010	7511	−1.87	−2.33	−1.41	−0.072	−0.145	0.002

According to WHO standards, total anaemia was defined as Hb < 12 g/dL for non-pregnant women aged 15–49 years; severe anaemia was defined as Hb < 8 g/dL. We used above repeated cross-sectional dataset (The four dataset) for the analyses of the annualized changes. All estimates used to determine anemia were weighted and proved appropriate for the DHS complex survey design. The changes in the prevalences of total and severe anemia were measured by the annual absolute change in percentage points and were estimated through a calculation of the difference between anemia prevalence in the earliest and most recent surveys divided by the number of years between these two surveys.

© 2018 Yang F et al. *JAMA Network Open*.

eTable 5. The Absolute and Relative Inequality of Total Anemia Among Children for Each Country at the Most Recent Survey

Country	SII of total anaemia			RII of total anaemia		
	SII	95%Confidence Interval		RII	95%Confidence Interval	
Albania	-13.88	-20.77	-7.00	0.40	0.25	0.63
Armenia	11.26	0.96	21.56	1.67	1.04	2.68
Azerbaijan	-13.82	-21.79	-5.86	0.55	0.39	0.78
Bangladesh	-21.67	-28.61	-14.74	0.42	0.31	0.55
Benin	-18.34	-24.32	-12.36	0.47	0.36	0.60
Bolivia	-21.33	-27.94	-14.71	0.41	0.31	0.54
Burkina Faso	-9.91	-12.71	-7.12	0.37	0.28	0.49
Burundi	-11.42	-17.15	-5.68	0.63	0.50	0.79
Cambodia	-21.66	-27.21	-16.11	0.41	0.33	0.52
Cameroon	-15.86	-20.89	-10.84	0.51	0.41	0.63
Congo Dem	-9.16	-16.37	-1.95	0.67	0.48	0.92
Congo	-13.34	-17.15	-9.52	0.56	0.48	0.66
Cote d'Ivoire	-23.01	-28.63	-17.40	0.28	0.21	0.39
Egypt	-18.61	-22.88	-14.33	0.40	0.32	0.49
Ethiopia	-18.01	-21.26	-14.75	0.48	0.42	0.55
Gabon	-13.39	-19.45	-7.32	0.56	0.43	0.73
Gambia	-23.24	-28.89	-17.58	0.30	0.22	0.41
Ghana	-35.96	-42.19	-29.74	0.18	0.13	0.25
Guinea	-18.00	-23.33	-12.68	0.36	0.27	0.49
Guyana	0.00	-8.45	8.45	1.00	0.70	1.42
Haiti	1.00	-4.58	6.58	1.05	0.82	1.34
Honduras	-10.62	-14.03	-7.20	0.60	0.51	0.71
Jordan	-16.28	-20.71	-11.84	0.47	0.38	0.58
Kyrgyzstan	-6.99	-12.69	-1.29	0.75	0.60	0.95
Lesotho	-4.38	-12.69	3.94	0.79	0.59	1.08
Madagascar	-16.00	-20.71	-11.30	0.53	0.43	0.64
Malawi	-17.38	-22.51	-12.25	0.47	0.37	0.59
Mali	-25.49	-29.44	-21.54	0.17	0.13	0.23
Moldova	-18.58	-27.24	-9.91	0.41	0.27	0.62
Mozambique	-28.46	-33.20	-23.73	0.27	0.21	0.33
Namibia	-14.36	-23.19	-5.53	0.56	0.39	0.80
Nepal	-7.68	-14.74	-0.62	0.73	0.55	0.98
Niger	-5.51	-9.73	-1.29	0.75	0.59	0.93
Peru	-27.82	-32.41	-23.22	0.30	0.26	0.35

© 2018 Yang F et al. *JAMA Network Open*.

Rwanda	-14.39	-19.97	-8.81	0.53	0.42	0.68
Sao Tome	-13.13	-21.76	-4.51	0.58	0.40	0.83
Senegal	-26.06	-30.95	-21.17	0.33	0.27	0.41
Sierra Leone	-6.34	-10.46	-2.22	0.67	0.51	0.87
Swaziland	3.57	-4.32	11.46	1.16	0.84	1.59
Tanzania	-4.72	-10.81	1.37	0.94	0.79	1.10
Timor-Leste	-0.63	-7.66	6.41	0.97	0.73	1.30
Togo	-8.40	-14.01	-2.79	0.67	0.51	0.87
Uganda	-17.77	-25.34	-10.19	0.36	0.26	0.51
Yemen	-14.26	-17.91	-10.61	0.24	0.17	0.35
Zimbabwe	-2.51	-8.17	3.16	0.90	0.71	1.14

According to WHO standards, total anaemia was defined as Hb < 11 g/dL for children aged 6-59 months; severe anaemia was defined as Hb < 7 g/dL. We used the above cross-sectional dataset (the first dataset) for the estimates of education-related absolute and relative inequality among children women. All estimates used to determine anemia were weighted and proved appropriate for the DHS complex survey design. The slope index of inequality (SII) and the relative index of inequality (RII) were calculated to determine the absolute and relative socioeconomic inequalities of anemia, respectively. SII > 0 and RII > 1 would indicate that individuals with lower socioeconomic status would be more likely to suffer from anemia, whereas the reverse inequality would indicate lower anemia prevalence among populations with lower socioeconomic status.

eTable 6. The Absolute and Relative Inequality of Total Anemia Among Nonpregnant Women for Each Country at the Most Recent Survey

Country	SII of total anaemia			RII of total anaemia		
	SII	95%Confidence Interval		RII	95%Confidence Interval	
Albania	-10.48	-13.46	-7.49	0.49	0.40	0.60
Armenia	1.68	-2.22	5.59	1.10	0.88	1.37
Azerbaijan	-12.06	-15.88	-8.24	0.59	0.50	0.70
Bangladesh	-19.96	-24.47	-15.45	0.44	0.36	0.53
Benin	-0.61	-5.70	4.48	0.98	0.79	1.20
Bolivia	-14.09	-18.49	-9.70	0.54	0.45	0.66
Burkina Faso	-13.16	-17.09	-9.23	0.59	0.50	0.69
Burundi	-9.49	-13.22	-5.76	0.50	0.38	0.66
Cambodia	-24.70	-28.18	-21.21	0.36	0.31	0.42
Cameroon	8.79	4.74	12.83	1.45	1.22	1.72
Congo	9.54	4.64	14.45	1.47	1.20	1.79
Congo Dem	0.48	-3.19	4.15	1.02	0.88	1.19
Cote d'Ivoire	-6.55	-11.87	-1.24	0.77	0.62	0.95
Egypt	-5.95	-9.40	-2.50	0.72	0.60	0.87

© 2018 Yang F et al. *JAMA Network Open*.

Ethiopia	-14.92	-16.86	-12.99	0.38	0.33	0.43
Gabon	9.51	4.77	14.26	1.48	1.22	1.81
Gambia	-26.40	-31.43	-21.36	0.33	0.26	0.41
Ghana	-8.52	-13.51	-3.54	0.70	0.57	0.86
Guinea	-20.21	-25.44	-14.98	0.44	0.36	0.55
Guyana	4.92	0.22	9.61	1.24	1.01	1.52
Haiti	12.98	9.34	16.62	1.68	1.45	1.95
Honduras	5.73	4.02	7.44	1.55	1.36	1.77
Jordan	0.60	-3.84	5.03	1.03	0.85	1.24
Kyrgyzstan	-12.25	-16.08	-8.42	0.58	0.49	0.69
Lesotho	6.41	1.54	11.29	1.39	1.08	1.79
Madagascar	-20.31	-23.83	-16.79	0.41	0.35	0.48
Malawi	-8.57	-12.44	-4.70	0.65	0.54	0.79
Mali	-18.58	-23.47	-13.69	0.47	0.39	0.58
Moldova	-11.19	-14.86	-7.52	0.57	0.47	0.68
Mozambique	-11.09	-14.18	-8.00	0.64	0.57	0.72
Namibia	-4.02	-8.42	0.38	0.78	0.60	1.02
Nepal	-2.59	-6.56	1.38	0.89	0.74	1.07
Niger	-11.76	-16.56	-6.96	0.62	0.51	0.75
Peru	-6.81	-8.64	-4.98	0.64	0.56	0.72
Rwanda	-7.86	-11.14	-4.57	0.60	0.48	0.74
Sao Tome	-4.99	-12.11	2.13	0.81	0.61	1.09
Senegal	-9.05	-14.15	-3.95	0.70	0.57	0.85
Sierra Leone	-19.73	-23.74	-15.72	0.45	0.38	0.53
Swaziland	7.60	2.99	12.21	1.44	1.15	1.79
Tanzania	5.57	2.03	9.12	1.26	1.09	1.45
Timor-Leste	-4.20	-8.88	0.48	0.78	0.59	1.03
Togo	19.72	14.82	24.62	2.23	1.82	2.73
Uganda	-16.30	-22.71	-9.89	0.50	0.38	0.66
Yemen	-12.85	-16.62	-9.09	0.51	0.42	0.62
Zimbabwe	1.82	-1.72	5.35	1.09	0.92	1.30

According to WHO standards, total anaemia was defined as Hb < 12 g/dL for non-pregnant women aged 15–49 years; severe anaemia was defined as Hb < 8 g/dL. We used the above cross-sectional dataset (the second dataset) for the estimates of absolute and relative inequality among non-pregnant women. All estimates used to determine anemia were weighted and proved appropriate for the DHS complex survey design. The slope index of inequality (SII) and the relative index of inequality (RII) were calculated to determine the absolute and relative socioeconomic inequalities of anemia, respectively. SII > 0 and RII > 1 would indicate that individuals with lower socioeconomic status would be more likely to suffer from anemia, whereas the reverse inequality would indicate lower anemia prevalence among populations with lower socioeconomic status.

© 2018 Yang F et al. *JAMA Network Open*.

eTable 7. The Annualized Changes of the Absolute and Relative Inequality of Total Anemia Among Children Between the Earliest and Most Recent Survey for Each Country

Country	SII of total anaemia			RII of total anaemia		
	The annualized change	95%Confidence Interval		The annualized % change	95%Confidence Interval	
Armenia	7.23	4.72	9.73	37.93	25.17	50.68
Benin	0.28	-0.57	1.13	5.51	1.13	9.89
Bolivia	-0.47	-2.31	1.37	-2.74	-10.41	4.94
Cambodia	-0.39	-1.35	0.56	-1.07	-5.15	3.01
Cameroon	0.48	-0.61	1.56	3.12	-1.78	8.02
Egypt	-0.16	-0.91	0.59	-2.52	-5.90	0.86
Ethiopia	-0.60	-1.63	0.44	-2.31	-6.54	1.91
Ghana	-1.30	-2.03	-0.58	-5.14	-8.91	-1.37
Guinea	-1.17	-2.30	-0.04	-7.08	-13.26	-0.90
Haiti	-0.22	-0.92	0.49	-0.88	-3.89	2.14
Honduras	0.54	-0.30	1.38	1.34	-2.46	5.15
Jordan	-1.04	-2.44	0.35	-5.65	-11.96	0.66
Lesotho	-4.15	-6.56	-1.74	-16.65	-26.34	-6.95
Madagascar	-0.49	-2.34	1.36	-0.07	-8.21	8.08
Malawi	-0.61	-1.98	0.76	-0.92	-7.48	5.64
Mali	-1.16	-1.74	-0.58	-8.54	-12.42	-4.66
Nepal	-0.78	-2.49	0.94	-3.15	-10.05	3.76
Niger	0.75	-0.25	1.74	6.17	0.21	12.13
Peru	-1.10	-1.74	-0.46	-5.49	-8.22	-2.75
Rwanda	-0.61	-1.48	0.26	-3.01	-6.66	0.64
Senegal	-2.00	-2.87	-1.13	-6.29	-10.93	-1.65
Sierra Leone	0.33	-1.16	1.82	0.78	-8.09	9.66
Tanzania	1.95	1.05	2.84	9.80	5.80	13.80
Zimbabwe	-0.39	-2.02	1.24	-1.60	-8.32	5.12

According to WHO standards, total anaemia was defined as Hb < 11 g/dL for children aged 6-59 months; severe anaemia was defined as Hb < 7 g/dL. We used above repeated cross-sectional dataset (The third dataset) for the analyses of the annualized changes. All estimates used to determine anemia were weighted and proved appropriate for the DHS complex survey design. The annualized changes of the SII and RII were calculated to adjust for the interval of time between the earliest and most recent surveys. Positive values for the annualized change of the SII and RII indicate a reduction in inequality, whereas negative values indicate an increase in inequality.

© 2018 Yang F et al. *JAMA Network Open*.

eTable 8. The Annualized Changes of the Absolute and Relative Inequality of Total Anemia Among Nonpregnant Women Between the Earliest and Most Recent Survey for Each Country

Country	SII of total anaemia			RII of total anaemia		
	The annualized change	95%Confidence Interval		The annualized % change	95%Confidence Interval	
Armenia	2.25	1.28	3.22	19.03	12.13	25.92
Benin	-0.81	-1.62	0.00	-3.48	-6.90	-0.06
Bolivia	1.90	0.68	3.12	9.86	4.31	15.41
Cambodia	-0.36	-1.03	0.31	-1.41	-4.22	1.39
Cameroon	0.35	-0.58	1.29	1.63	-2.22	5.49
Congo	3.28	1.95	4.62	13.30	7.87	18.74
Egypt	-0.20	-0.81	0.41	-1.70	-4.57	1.17
Ethiopia	0.88	0.24	1.52	0.91	-2.84	4.65
Ghana	0.63	0.01	1.25	2.52	-0.03	5.07
Guinea	-1.49	-2.61	-0.37	-6.08	-10.62	-1.54
Haiti	0.87	0.34	1.40	3.49	1.34	5.63
Honduras	1.13	0.70	1.56	8.47	5.36	11.58
Jordan	-0.16	-1.52	1.21	-0.69	-6.51	5.14
Lesotho	-2.15	-3.66	-0.65	-9.54	-16.98	-2.11
Madagascar	1.73	0.22	3.23	6.30	-0.24	12.83
Malawi	-0.77	-2.04	0.50	-4.38	-10.08	1.32
Mali	0.06	-0.62	0.74	0.70	-2.17	3.57
Nepal	-0.54	-1.56	0.48	-2.51	-7.12	2.10
Niger	0.78	-0.39	1.96	3.26	-1.59	8.11
Peru	0.67	0.32	1.02	2.13	0.15	4.11
Rwanda	0.10	-0.47	0.67	-0.53	-3.90	2.83
Senegal	0.78	-0.76	2.32	3.43	-2.86	9.73
Sierra Leone	-3.67	-5.09	-2.25	-14.95	-20.77	-9.13
Tanzania	1.85	1.00	2.69	7.48	4.06	10.91
Zimbabwe	-0.33	-1.37	0.72	-1.22	-5.97	3.54

According to WHO standards, total anaemia was defined as Hb < 12 g/dL for non-pregnant women aged 15–49 years; severe anaemia was defined as Hb < 8 g/dL. We used above repeated cross-sectional dataset (The four dataset) for the analyses of the annualized changes. All estimates used to determine anemia were weighted and proved appropriate for the DHS complex survey design. The annualized changes of the SII and RII were calculated to adjust for the interval of time between the earliest and most recent surveys. Positive values for the annualized change of the SII and RII indicate a reduction in inequality, whereas negative values indicate an increase in inequality.

© 2018 Yang F et al. *JAMA Network Open*.

eTable 9. Education-Related Absolute and Relative Inequality of Total Anemia Among Children for Each Country at the Most Recent Survey

Country	SII of total anaemia			RII of total anaemia		
	SII	95%Confidence Interval		RII	95%Confidence Interval	
Albania	-11.25	-18.50	-3.99	0.48	0.30	0.77
Armenia	1.47	-8.66	11.60	1.07	0.67	1.70
Azerbaijan	-8.03	-15.83	-0.23	0.71	0.51	0.99
Bangladesh	-11.61	-18.67	-4.56	0.63	0.47	0.83
Benin	-11.29	-17.20	-5.39	0.63	0.49	0.80
Bolivia	-11.51	-18.26	-4.76	0.62	0.46	0.82
Burkina Faso	-6.62	-9.42	-3.81	0.52	0.40	0.69
Burundi	-6.43	-12.33	-0.53	0.77	0.60	0.98
Cambodia	-12.48	-18.06	-6.90	0.60	0.48	0.76
Cameroon	-15.70	-20.72	-10.69	0.51	0.41	0.63
Congo Dem	-6.62	-13.92	0.67	0.75	0.54	1.03
Congo	-4.61	-8.49	-0.74	0.82	0.69	0.97
Cote d'Ivoire	-9.72	-15.36	-4.08	0.59	0.44	0.80
Egypt	-10.88	-15.40	-6.35	0.59	0.47	0.73
Ethiopia	-11.87	-15.51	-8.24	0.62	0.54	0.72
Gabon	-13.72	-20.09	-7.35	0.55	0.42	0.73
Gambia	-12.78	-18.42	-7.14	0.51	0.38	0.69
Ghana	-31.64	-37.88	-25.40	0.22	0.16	0.30
Guinea	-12.99	-18.49	-7.49	0.49	0.36	0.66
Guyana	-2.31	-11.26	6.65	0.91	0.62	1.32
Haiti	-2.71	-8.26	2.85	0.89	0.69	1.13
Honduras	-6.37	-9.75	-2.98	0.74	0.63	0.87
Jordan	-8.53	-12.61	-4.46	0.67	0.56	0.81
Kyrgyzstan	5.52	-0.24	11.28	1.25	0.99	1.58
Lesotho	5.54	-7.96	19.04	0.89	0.58	1.37
Madagascar	-12.62	-17.39	-7.86	0.60	0.50	0.73
Malawi	-15.59	-20.67	-10.50	0.50	0.40	0.63
Mali	-15.18	-19.22	-11.15	0.37	0.28	0.48
Moldova	-13.68	-22.25	-5.10	0.52	0.34	0.78
Mozambique	-16.56	-21.26	-11.86	0.47	0.38	0.59
Namibia	-12.97	-21.60	-4.33	0.59	0.42	0.84
Nepal	-10.54	-17.92	-3.16	0.65	0.49	0.88
Niger	-1.76	-6.13	2.60	0.91	0.72	1.15
Peru	-17.00	-21.82	-12.18	0.51	0.43	0.60
Rwanda	-4.96	-10.65	0.73	0.81	0.63	1.03

© 2018 Yang F et al. *JAMA Network Open*.

Sao Tome	-11.51	-20.06	-2.96	0.62	0.43	0.88
Senegal	-8.51	-13.14	-3.89	0.70	0.57	0.85
Sierra Leone	-2.83	-7.03	1.37	0.84	0.64	1.09
Swaziland	-1.65	-9.59	6.30	0.94	0.68	1.29
Tanzania	-6.93	-12.06	-1.80	0.82	0.70	0.96
Timor-Leste	3.37	-3.64	10.38	1.15	0.86	1.53
Togo	-10.85	-16.64	-5.06	0.59	0.45	0.78
Uganda	-10.35	-18.54	-2.15	0.52	0.37	0.73
Zimbabwe	-6.14	-11.89	-0.39	0.78	0.61	0.98
Total	-8.37	-9.60	-7.14	0.71	0.68	0.75

According to WHO standards, total anaemia was defined as Hb < 11 g/dL for children aged 6-59 months; severe anaemia was defined as Hb < 7 g/dL. We used the above cross-sectional dataset (the first dataset) for the estimates of education-related absolute and relative inequality among children. All estimates used to determine anemia were weighted and proved appropriate for the DHS complex survey design. The slope index of inequality (SII) and the relative index of inequality (RII) were calculated to determine the absolute and relative socioeconomic inequalities of anemia, respectively. SII > 0 and RII > 1 would indicate that individuals with lower socioeconomic status would be more likely to suffer from anemia, whereas the reverse inequality would indicate lower anemia prevalence among populations with lower socioeconomic status.

© 2018 Yang F et al. *JAMA Network Open*.

eTable 10. Education-Related Absolute and Relative Inequality of Total Anemia Among Nonpregnant Women for Each Country at the Most Recent Survey

Country	SII of total anaemia			RII of total anaemia		
	SII	95%Confidence Interval		RII	95%Confidence Interval	
Albania	-7.67	-10.72	-4.62	0.59	0.48	0.73
Armenia	1.77	-2.00	5.54	1.10	0.89	1.36
Azerbaijan	-4.36	-8.05	-0.66	0.83	0.71	0.97
Bangladesh	-13.22	-17.79	-8.65	0.58	0.48	0.70
Benin	-0.67	-5.65	4.31	0.97	0.79	1.20
Bolivia	-7.40	-11.83	-2.98	0.73	0.60	0.88
Burkina Faso	-7.92	-11.79	-4.04	0.73	0.62	0.85
Burundi	-8.76	-12.61	-4.91	0.53	0.40	0.70
Cambodia	-15.18	-18.68	-11.68	0.54	0.47	0.62
Cameroon	3.50	-0.57	7.57	1.16	0.98	1.38
Congo	5.29	0.30	10.28	1.24	1.01	1.51
Congo Dem	3.77	0.07	7.48	1.17	1.00	1.37
Cote d'Ivoire	-8.18	-13.50	-2.86	0.72	0.58	0.89
Egypt	-1.28	-4.91	2.34	0.93	0.77	1.14
Ethiopia	-16.79	-18.94	-14.63	0.33	0.29	0.38
Gabon	7.86	2.85	12.87	1.38	1.12	1.70
Gambia	-18.11	-23.28	-12.94	0.47	0.37	0.58
Ghana	-5.33	-10.31	-0.34	0.80	0.65	0.99
Guinea	-13.64	-18.90	-8.38	0.58	0.47	0.71
Guyana	-4.15	-9.06	0.75	0.84	0.68	1.03
Haiti	8.02	4.35	11.69	1.38	1.19	1.60
Honduras	1.71	0.01	3.41	1.14	1.00	1.30
Jordan	-2.27	-6.31	1.78	0.91	0.77	1.08
Kyrgyzstan	2.18	-1.62	5.97	1.10	0.93	1.30
Lesotho	2.45	-2.46	7.35	1.13	0.88	1.46
Madagascar	-19.52	-23.11	-15.94	0.42	0.36	0.50
Malawi	-9.95	-13.80	-6.11	0.61	0.51	0.74
Mali	-14.04	-19.02	-9.06	0.57	0.47	0.70
Moldova	-3.69	-7.30	-0.07	0.83	0.69	1.00
Mozambique	-8.65	-11.68	-5.62	0.71	0.63	0.80
Namibia	-6.18	-10.54	-1.82	0.68	0.52	0.89
Nepal	-4.69	-8.82	-0.56	0.81	0.67	0.97
Niger	-8.44	-13.39	-3.49	0.71	0.58	0.87

© 2018 Yang F et al. *JAMA Network Open*.

Peru	-5.12	-6.86	-3.38	0.71	0.64	0.80
Rwanda	-5.45	-8.80	-2.10	0.70	0.56	0.87
Sao Tome	1.49	-5.61	8.59	1.06	0.79	1.42
Senegal	0.17	-4.62	4.95	1.01	0.83	1.22
Sierra Leone	-10.92	-14.93	-6.91	0.64	0.55	0.76
Swaziland	-1.82	-6.49	2.85	0.92	0.73	1.15
Tanzania	0.57	-2.80	3.95	1.02	0.89	1.18
Timor-Leste	-5.96	-10.60	-1.32	0.70	0.53	0.93
Togo	7.85	2.77	12.93	1.37	1.12	1.69
Uganda	-14.12	-20.71	-7.53	0.55	0.42	0.73
Zimbabwe	0.42	-3.16	3.99	1.02	0.86	1.21
Total	-4.88	-5.46	-4.29	0.80	0.78	0.83

According to WHO standards, total anaemia was defined as Hb < 12 g/dL for non-pregnant women aged 15–49 years; severe anaemia was defined as Hb < 8 g/dL. We used the above cross-sectional dataset (the second dataset) for the estimates of education-related absolute and relative inequality among non-pregnant women. All estimates used to determine anemia were weighted and proved appropriate for the DHS complex survey design. The slope index of inequality (SII) and the relative index of inequality (RII) were calculated to determine the absolute and relative socioeconomic inequalities of anemia, respectively. SII > 0 and RII > 1 would indicate that individuals with lower socioeconomic status would be more likely to suffer from anemia, whereas the reverse inequality would indicate lower anemia prevalence among populations with lower socioeconomic status.

© 2018 Yang F et al. *JAMA Network Open*.

eTable 11. The Annualized Changes of Education-Related Absolute and Relative Inequality of Total Anemia Among Children Between the Earliest and Most Recent Survey for Each Country

Country	SII of total anaemia			RII of total anaemia		
	The annualized change	95%Confidence Interval		The annualized % change	95%Confidence Interval	
Armenia	3.22	0.73	5.70	17.12	4.75	29.48
Benin	0.68	-0.17	1.53	6.51	2.17	10.84
Bolivia	0.85	-0.99	2.69	2.99	-4.62	10.60
Cambodia	0.84	-0.14	1.81	4.05	-0.08	8.18
Cameroon	-0.25	-1.33	0.83	-0.33	-5.19	4.54
Egypt	0.75	-0.03	1.53	1.95	-1.55	5.44
Ethiopia	-0.77	-1.89	0.36	-3.03	-7.57	1.51
Ghana	-1.40	-2.13	-0.66	-5.86	-9.63	-2.08
Guinea	-1.62	-2.76	-0.49	-9.09	-15.23	-2.94
Haiti	-0.02	-0.71	0.67	-0.13	-3.09	2.82
Honduras	1.34	0.51	2.17	5.16	1.42	8.89
Jordan	0.16	-1.11	1.43	0.14	-5.53	5.81
Lesotho	-2.10	-4.94	0.74	-8.43	-19.79	2.94
Madagascar	0.54	-1.36	2.44	4.27	-4.06	12.59
Malawi	-1.64	-3.01	-0.27	-6.49	-13.00	0.02
Mali	-0.78	-1.36	-0.20	-5.32	-9.10	-1.54
Nepal	0.72	-1.06	2.49	2.86	-4.32	10.04
Niger	0.90	-0.13	1.93	6.26	0.15	12.37
Peru	-0.13	-0.76	0.50	-1.00	-3.67	1.66
Rwanda	0.43	-0.46	1.31	1.52	-2.17	5.21
Senegal	0.19	-0.64	1.02	3.60	-0.82	8.02
Sierra Leone	0.70	-0.81	2.21	3.41	-5.57	12.39
Tanzania	0.27	-0.60	1.13	1.99	-1.84	5.83
Zimbabwe	-1.40	-3.03	0.23	-5.76	-12.50	0.98

According to WHO standards, total anaemia was defined as Hb < 11 g/dL for children aged 6-59 months; severe anaemia was defined as Hb < 7 g/dL. We used above repeated cross-sectional dataset (The third dataset) for the analyses of the annualized changes. All estimates used to determine anemia were weighted and proved appropriate for the DHS complex survey design. The annualized changes of the SII and RII were calculated to adjust for the interval of time between the earliest and most recent surveys. Positive values for the annualized change of the SII and RII indicate a reduction in education-related inequality, whereas negative values indicate an increase in education-related inequality.

© 2018 Yang F et al. *JAMA Network Open*.

eTable 12. The Annualized Changes of Education-Related Absolute and Relative Inequality of Total Anemia Among Nonpregnant Women Between the Earliest and Most Recent Survey for Each Country

Country	SII of total anaemia			RII of total anaemia		
	The annualized change	95%Confidence Interval		The annualized % change	95%Confidence Interval	
Armenia	0.81	-0.15	1.78	6.01	-0.73	12.75
Benin	-0.77	-1.57	0.04	-3.29	-6.68	0.11
Bolivia	1.05	-0.17	2.27	5.27	-0.18	10.72
Cambodia	-0.22	-0.90	0.45	-0.89	-3.67	1.90
Cameroon	-1.10	-2.03	-0.17	-4.40	-8.25	-0.55
Congo	1.94	0.61	3.28	7.86	2.48	13.24
Egypt	0.21	-0.42	0.84	0.67	-2.31	3.65
Ethiopia	-0.24	-0.95	0.46	-5.42	-9.51	-1.33
Ghana	0.56	-0.07	1.19	2.26	-0.32	4.83
Guinea	-1.56	-2.67	-0.45	-6.29	-10.77	-1.81
Haiti	1.09	0.56	1.62	4.37	2.25	6.50
Honduras	0.74	0.31	1.17	5.26	2.14	8.39
Jordan	0.57	-0.67	1.82	2.52	-2.80	7.84
Lesotho	-0.74	-2.26	0.77	-3.25	-10.73	4.23
Madagascar	1.52	0.01	3.04	5.57	-1.00	12.13
Malawi	-0.27	-1.53	0.99	-2.55	-8.20	3.11
Mali	0.05	-0.63	0.73	0.55	-2.30	3.39
Nepal	0.87	-0.18	1.92	3.67	-1.09	8.42
Niger	-0.44	-1.65	0.77	-1.79	-6.75	3.17
Peru	0.76	0.43	1.10	2.84	0.96	4.72
Rwanda	0.20	-0.38	0.78	0.34	-3.08	3.75
Senegal	2.57	1.11	4.03	10.66	4.69	16.62
Sierra Leone	-2.16	-3.60	-0.72	-8.77	-14.61	-2.92
Tanzania	1.36	0.56	2.16	5.45	2.21	8.69
Zimbabwe	0.34	-0.71	1.38	1.49	-3.29	6.28

According to WHO standards, total anaemia was defined as Hb < 12 g/dL for non-pregnant women aged 15–49 years; severe anaemia was defined as Hb < 8 g/dL. We used above repeated cross-sectional dataset (The four dataset) for the analyses of the annualized changes. All estimates used to determine anemia were weighted and proved appropriate for the DHS complex survey design. The annualized changes of the SII and RII were calculated to adjust for the interval of time between the earliest and most recent surveys. Positive values for the annualized change of the SII and RII indicate a reduction in education-related inequality, whereas negative values indicate an increase in education-related inequality.

© 2018 Yang F et al. *JAMA Network Open*.
